# Ionizing radiation: molecular mechanisms, biological effects, and therapeutic targets

**DOI:** 10.1186/s43556-025-00358-4

**Published:** 2026-01-09

**Authors:** Wei Wei, Yifan Ren, Jinxin Lan, Junxuan Yi, Mingwei Wang, Yadi Zhang, Shuyuan Wang, Yinmei Xu, Guiqiao Han, Yankan Fu, Lukuan You, Junxia Xue, Shunzi Jin, Jianxiong Li

**Affiliations:** 1 School of Medicine, Nankai University, Tianjin, 300071 China; 2 Senior Department of Oncology, Chinese PLA General Hospital, 100853 Beijing, China; 3 Department of Neurosurgery, Xuanwu Hospital, China International Neuroscience Institute, Capital Medical University, 45 Changchun St, Beijing, 100053 China; 4 Department of Radiation Oncology, Cancer Center, Affiliated Dongguan Hospital, Southern Medical University, Guangdong, China; 5 NHC Key Laboratory of Radiobiology, School of Public Health, Jilin University, Changchun, 130021 China

**Keywords:** Ionizing radiation, Oxidative stress, Epigenetics, Organ dysfunction, Signal pathway, Therapeutic targets

## Abstract

Radiation-induced injury remains a significant challenge in the radiotherapy of cancer patients. Ionizing radiation causes various cellular and molecular damages, leading to both acute and chronic organ dysfunction. Its impact extends beyond interrupting standard treatment protocols and adversely affects the quality of life. Therefore, understanding the mechanisms underlying radiation-induced injury and identifying effective treatment strategies are crucial. In this review, we summarize the recent advances in the molecular and cellular mechanisms of radiation-induced injury across various organs and systems, particularly in the lung, gastrointestinal system, brain, skin, and bone. We highlight the roles of oxidative stress, DNA damage response, mitochondrial dysfunction, and epigenetics in radiation pathology, and summarize the relevant signaling pathways and cellular responses involved in radiation damage. Additionally, we discuss the common symptoms, risk factors, and current diagnostic strategies of radiation-induced injuries. Furthermore, this article provides an in-depth review of effective clinical treatments, elucidates their mechanisms of action, and highlights emerging therapeutic approaches, such as stem cell therapy, nanomedicine, and exosome-based interventions, in clinical practice. Despite significant advances in understanding radiation-induced injury, challenges remain in translating molecular insights into effective therapies. The review concludes with a call for integrated, precision medicine-based approaches to better manage radiation-induced injuries and improve patient outcomes.

## Background

Radiotherapy has been a cornerstone of cancer treatment since its introduction in the early twentieth century, quickly becoming a critical approach for managing various cancers. This treatment utilizes ionizing radiation (IR), such as X-rays, γ-rays, and particle radiation, to target and destroy tumor cells. However, while effective against cancerous tissues, radiotherapy inevitably exposes surrounding healthy tissues to radiation, leading to a range of complications known as radiation-induced injuries. IR, in its various forms, exerts distinct mechanisms of action on tissues. High-energy photons (X-rays and γ-rays) primarily induce cellular damage indirectly by generating reactive oxygen species (ROS), which cause DNA strand breaks and cellular dysfunction. In contrast, particle radiation, such as protons and carbon ions, has a higher linear energy transfer (LET), resulting in more direct DNA damage and complex cellular lesions that are more difficult to repair. These differences in radiation quality influence the severity and nature of radiation-induced injuries [1]. Tissue sensitivity to radiation varies, with the following general order of sensitivity: bone marrow tissue, gastrointestinal epithelium, skin, vascular endothelium, alveolar epithelium, central nervous system (CNS), bone and cartilage, and connective tissue. The varying response of tissues to radiation, along with radiation type and treatment protocols, leads to a broad spectrum of radiation-induced injuries [2].

Radiation-induced injuries can significantly complicate treatment, worsening patients' conditions and disrupting the planned course of therapy, often leading to poorer prognoses. For example, patients suffering from acute cerebrovascular syndromes may die within hours to days, and those with severe gastrointestinal complications may pass within a week [3]. Given the complexity and overlap of radiation-induced injuries with tumor recurrence, clinical manifestations are often ambiguous, making early diagnosis and timely intervention critical. Current diagnostic approaches rely heavily on clinical assessments, physical exams, imaging results, and laboratory findings, but these methods are mostly subjective and can delay the initiation of appropriate treatment [4].

In light of these challenges, there is a pressing need for improved diagnostic tools and more effective therapeutic strategies. Understanding the intricate molecular mechanisms, cellular pathways, and tissue-specific responses involved in radiation-induced injury is crucial for developing targeted interventions. In this review, we first summarize the cellular and molecular mechanisms, focusing on oxidative stress, DNA damage response, and epigenetic regulation. Then, we describe the cellular responses and functional changes at both systemic and organ-specific levels, covering the immune system, hematopoietic system, brain, lung, cardiovascular system, gastrointestinal system, bone, skin, and reproductive system. Next, we evaluate the risk factors, clinical manifestations, long-term sequelae, and diagnostic methods of radiation exposure, integrating epidemiological findings with mechanistic data. Finally, we discuss recent advancements in targeted therapeutic agents, emphasizing their potential in minimizing radiation-induced injury while preserving therapeutic efficacy. This review aims to enhance the understanding of radiation-induced injury and improve clinical outcomes for cancer patients undergoing radiotherapy, ultimately offering hope for better quality of life and extended survival.

## Molecular mechanisms of ionizing radiation-induced damage

IR exposure can cause various biological effects in cells and tissues. These effects are both direct, involving the damage of molecular components, and indirect, primarily through the generation of ROS. Radiation can induce a range of damage in tissues, such as double-strand breaks (DSBs) in DNA, disruption of organelles, and changes in cellular signaling pathways.

### Direct DNA damage

IR induces direct DNA damage through the formation of single-strand breaks (SSBs) and DSBs, both of which are critical for the integrity of the genome. DSBs are considered the most lethal form of DNA damage as they can lead to chromosomal fragmentation and genome instability. DNA damage activates the DNA damage response (DDR) pathway, which involves key players such as Ataxia Telangiectasia Mutated (ATM), Ataxia Telangiectasia and Rad3-related (ATR), and DNA-dependent protein kinase (DNA-PK). These molecules activate checkpoint kinases and transcription factors, such as p53. Depending on the severity of the damage, the cell either repairs the damage, arrests the cell cycle to allow time for repair, or undergoes apoptosis. If the damage is irreparable, cellular senescence may occur, contributing to tissue dysfunction and chronic inflammation [5].

While DNA repair mechanisms such as non-homologous end joining (NHEJ) and homologous recombination (HR) are essential for repairing these breaks, they are error-prone and can sometimes result in mutations. If improperly repaired, this can lead to tumorigenesis [6].

### Indirect damage via Reactive Oxygen Species (ROS)

The indirect effects of IR are mediated through the generation of ROS due to water radiolysis. These ROS, including superoxide (O_2_•-), hydrogen peroxide (H_2_O_2_), and hydroxyl radicals (•OH), can damage cellular components, such as lipids, proteins, and DNA [7]. ROS can also play a role in cellular signaling pathways, triggering apoptosis, necrosis, or senescence depending on the cellular context. ROS-induced oxidative stress activates various transcription factors, including Nuclear Factor Kappa-Light-Chain-Enhancer of Activated B Cells (NF-κB) and Activator Protein 1 (AP-1), which upregulate pro-inflammatory cytokines such as Tumor Necrosis Factor-alpha (TNF-α), Interleukin-6 (IL-6), and Transforming Growth Factor-beta (TGF-β). These cytokines further promote the inflammatory cascade. The damage and repair responses are interconnected and persist continuously, leading to tissue damage, fibrosis, and eventual organ dysfunction [8].

### Mitochondrial DNA (mtDNA) damage

Following IR, radiolysis of water and disrupted mitochondrial respiration generate ROS that oxidize mtDNA. Lacking protective histones and possessing limited base-excision repair, mtDNA accumulates lesions, deletions, and replication stalls that impair oxidative phosphorylation and amplify ROS in a feed-forward loop. Damaged mitochondria undergo fission and mitophagy for clearance, but if this process is insufficient, the mitochondrial permeability transition pore (mPTP) or BCL2-associated X protein/BCL2 antagonist/killer (BAX/BAK) macropores open, allowing mtDNA to escape into the cytosol [9]. Cytosolic mtDNA levels rise within hours and can persist for days, activating cyclic GMP-AMP Synthase-Stimulator of Interferon Genes (cGAS-STING) to induce type-I interferon and NF-κB programs that link acute injury to chronic inflammation and senescence. Additionally, mtDNA engages endolysosomal nucleic-acid sensors and promotes inflammasome activation [10, 11]. Thus, IR-induced mtDNA damage impacts cells both intrinsically, by disrupting bioenergetics, and extrinsically, by acting as a damage-associated molecular pattern that propagates bystander effects and promotes fibrogenic immunity across organs.

### Epigenetic regulation

IR reprograms chromatin and the transcriptome beyond direct DNA breaks. Key epigenetic modifications include DNA methylation, histone variant/modification remodeling, RNA modifications, and non-coding RNAs, together amplifying inflammation, senescence, and fibroblast activation, contributing to long-term organ dysfunction [12, 13].

DNA methylation. IR alters CpG methylation in normal tissues and blood, with epigenome-wide studies identifying differentially methylated regions (DMRs) associated with DNA repair, inflammation, and subsequent toxicity risk. Endothelial cells exhibit dose-responsive methylation shifts even at low doses, implicating vascular epigenetics in late injury. Differential methylation in radiation-induced heart disease (RIHD) is linked to pathways related to its pathogenesis, such as endothelial activation, oxidative stress, and inflammation [12].

Histone modifications/chromatin. Profibrotic signaling is reinforced by Polycomb Repressive Complex 2/Enhancer of Zeste Homolog (PRC2/EZH2)- and G9a-driven methylation (Histone H3 Lysine 27 Trimethylation/Histone H3 Lysine 9 Di/Trimethylation (H3K27me3/H3K9me2/3)) and by p300-dependent acetylation, which together promote TGF-β and STAT3 programs in fibrotic organs. G9a-mediated histone methylation influences radiation-induced epithelial-mesenchymal transition (EMT). Metabolites like lactic acid can lactylate histones, promoting pro-fibrotic gene expression in lung macrophages, contributing to radiation-induced pulmonary fibrosis (RIPF) [14]. In addition, IR also induces senescence-associated chromatin states. For example, the histone variant H2A.J accumulates in keratinocytes after IR and tunes senescence-associated secretory phenotype (SASP), which participates in the development of radiation dermatitis (RD) together with the epigenetic regulation of TGF-β-driven myofibroblast program [15].

RNA methylation, particularly N6-methyladenosine (m6A) and 5-methylcytosine (m5C), plays a pivotal role in radiation-induced injury. Multiple studies have highlighted m6A as central to fibrogenesis. Radiation increases Methyltransferase 3 (METTL3) expression, enhancing YTH N6-Methyladenosine RNA Binding Protein 2 (YTHDF2)-dependent repression of Forkhead Box O1 (FOXO1), which promotes EMT and fibroblast activation. Additionally, METTL3 modifies Yin Yang 1 (YY1) to sustain profibrotic transcription. The demethylase activity of AlkB Homolog 5 (ALKBH5) exhibits a ‘double-edged sword’ effect. In the liver, ALKBH5-mediated demethylation of TIR domain-containing adaptor protein (TIRAP) mRNA activates NF-κB and c-Jun N-terminal Kinase/Mothers Against Decapentaplegic Homolog 2 (JNK/Smad2) signaling, thereby promoting radiation-induced hepatic fibrosis; in contrast, in radiation-induced lung injury (RILI), evidence suggests that ALKBH5 suppresses inflammation by demethylating IL-6 mRNA [16, 17].

Non-coding RNAs (ncRNAs), including microRNAs (miRNAs) and long non-coding RNAs (lncRNAs), play pivotal roles in the cellular response to radiation-induced damage [18]. For instance, lncRNA PVT1 acts as a competitive endogenous RNA (ceRNA) by sponging miR-9-5p, thereby activating the Mitogen-Activated Protein Kinase (MAPK) signaling pathway and exacerbating vascular endothelial cell injury after radiation. Similarly, lncRNA LIRR1 is upregulated in lung tissues post-radiation and mediates DNA damage response signaling in a p53-dependent manner, influencing radiosensitivity. Many studies have confirmed the role of ncRNAs in radiation-induced brain injury (RIBI), RILI, and radiation-induced gonadal injury [19–22].

### Radiation-induced signaling pathways

**Fig. 1:**
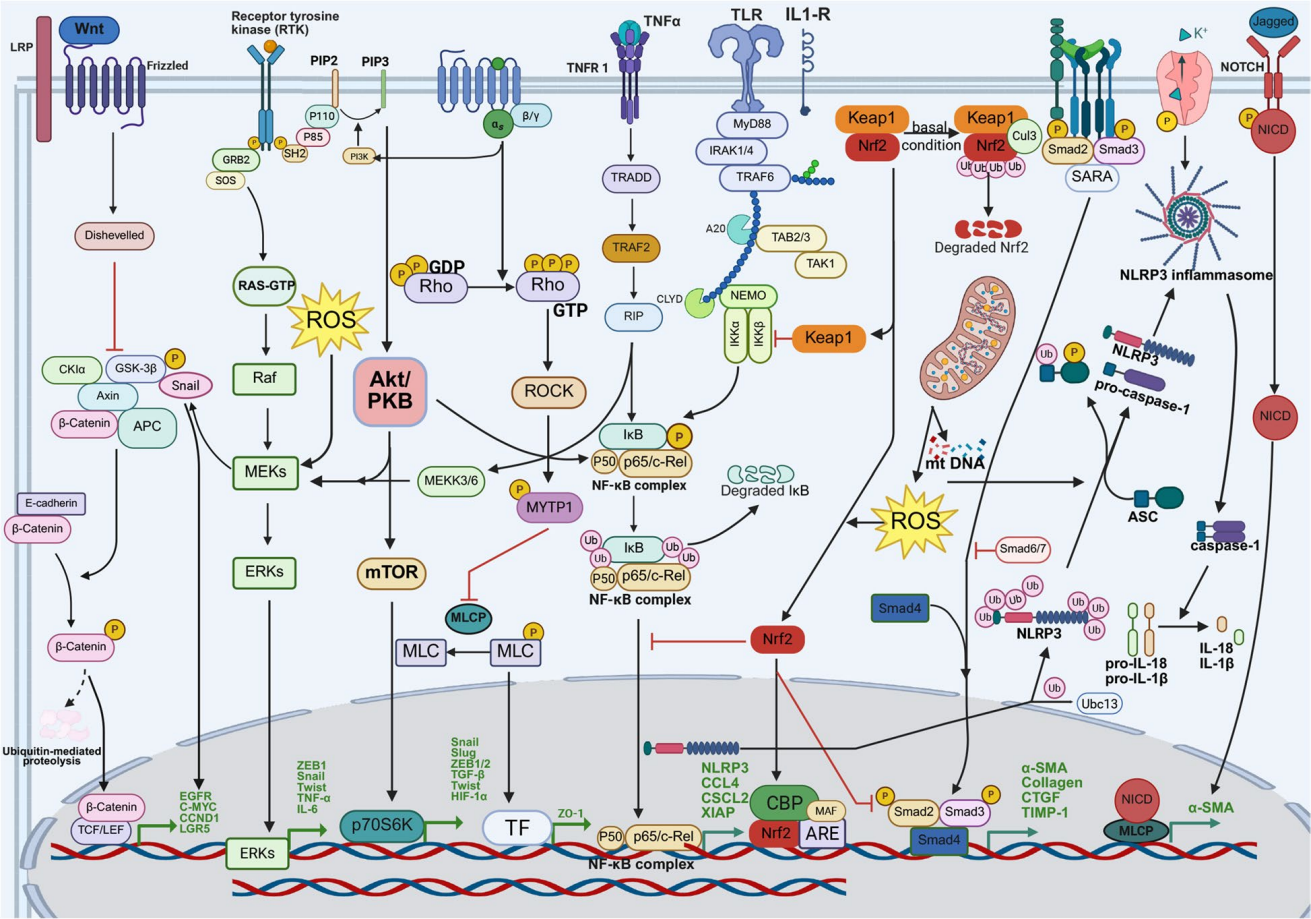
Key Cellular Pathways in Radiation-induced injury

Radiation-induced injury triggers complex cellular responses through various signaling pathways that govern tissue damage, inflammation, repair, and fibrosis. These interconnected signaling networks determine the severity of radiation-induced injury and the success of tissue repair, influencing both acute and chronic outcomes across various organ systems (Fig. 1). Understanding these pathways provides critical insights for developing targeted therapeutic strategies to mitigate radiation-induced injury.

#### Inflammation and profibrotic remodeling

##### Transforming Growth Factor Beta (TGF-β) Related Pathways

IR activates latent TGF-β through ROS-mediated cleavage and integrin-dependent mechanisms for release. Activated TGF-β binds TGF-β Receptor Type II (TβRII), which recruits and phosphorylates TGF-β Receptor Type I (TβRI), leading to phosphorylation of Smad2/3 and inhibition of Smad7. The Smad2/3-Smad4 complex translocates to the nucleus, initiating transcription of extracellular matrix (ECM) genes such as Collagen Type I Alpha 1 Chain (COL1A1), fibronectin, and Alpha-Smooth Muscle Actin (α-SMA). Persistent activation promotes EMT and myofibroblast accumulation, which drives radiation fibrosis in multiple organs. In animal models, the heightened pathway activity is closely associated with radiotoxicity, and the attenuation of TGF-β/Smad signaling reduces fibrosis. In the lung, this cascade underlies the progression from pneumonitis to fibrosis, characterized histologically by alveolar wall thickening and excessive collagen deposition. In the esophagus, the activation of the TGF-β signaling pathway contributes to submucosal fibrosis and the development of late radiation-induced strictures. In the skin, it contributes to chronic RD (CRD) and dermal scarring. In muscle and soft tissues, TGF-β blockade can alleviate post-radiotherapy contracture. Once cross-regulated with ROS, MAPK, PI3K-AKT, and Wnt pathways, pro-fibrotic signals can be further amplified, which accelerates the progression of radiation-induced fibrosis [23, 24].

##### Jagged1/Notch Pathway

IR upregulates Notch ligands, particularly Jagged1 and Delta-like 4, which engage Notch receptors on fibroblasts, macrophages, and endothelial cells. Ligand binding triggers γ-secretase cleavage and nuclear translocation of the Notch intracellular domain (NICD), activating transcription factors Hairy and Enhancer of Split (Hes) and Hairy/Enhancer-of-split related with YRPW motif (Hey). These factors promote fibroblast activation and macrophage M2 polarization, sustaining a profibrotic environment. In the lung, Notch can also sustain basal stem-cell survival by regulating DNA-damage checkpoints, thereby shaping the progression from pneumonitis to fibrosis. Selective inhibition of this signaling pathway reduces the degree of fibrosis in lung tissue and modulates macrophage polarization. In irradiated skin, Notch drives dermal fibroblast proliferation and angiogenic remodeling, whereas in bone marrow, it alters stromal niches, aggravating long-term hematopoietic suppression. In the intestine, Notch interacts with Wnt signaling to regulate progenitor plasticity during crypt regeneration [25, 26].

##### Ras Homolog Family Member A/Rho-Associated Coiled-Coil Containing Protein Kinase (RhoA/ROCK) Pathway

Endothelial RhoA/ROCK signaling represents an early driver of vascular dysfunction after irradiation. RhoA activation following IR stimulates ROCK1/2, which phosphorylate myosin light chain, induce actin stress fiber assembly, and disrupt adherens junctions such as Vascular Endothelial cadherin (VE-cadherin). This leads to endothelial barrier dysfunction, vascular leakage, and leukocyte infiltration. In the heart, endothelial permeability mediated by RhoA/ROCK contributes to interstitial edema and subsequent myocardial fibrosis. In the central nervous system, the same pathway accelerates blood–brain barrier breakdown, predisposing to radiation-induced cerebral edema and white matter injury. In the lung, the inhibition of the RhoA/ROCK signaling pathway restores barrier integrity across experimental models and has been associated with reduced long-term cardiopulmonary remodeling following thoracic radiotherapy. These vascular alterations converge with TGF-β-mediated profibrotic signaling during the late stages of RILI [27, 28].

##### NLR Family Pyrin Domain Containing 3 (NLRP3) Inflammasome Pathways

Mitochondrial ROS, DNA release, and potassium efflux following IR activate the NLRP3 inflammasome complex, which recruits Apoptosis-Associated Speck-like Protein containing a CARD (ASC) and caspase-1. Activated caspase-1 cleaves pro-Interleukin-1 Beta (pro-IL-1β) and pro-Interleukin-18 (pro-IL-18) into their mature forms, initiating a strong inflammatory response and inducing pyroptosis. In the lung, NLRP3-driven cytokine release recruits neutrophils and macrophages, fueling the transition from acute pneumonitis to fibrosis. The inhibition of NLRP3 inflammasome can attenuate inflammatory infiltration and fibrotic remodeling. In the intestine, excessive inflammasome activation disrupts epithelial integrity, leading to barrier failure and bacterial translocation. Moreover, low-dose radiation can prime NLRP3 in myeloid cells, highlighting dose- and compartment-specific effects [29, 30].

#### Epithelial survival, regeneration, and stemness

##### Wingless-related integration site (Wnt)/β-catenin Pathway

Under basal conditions, β-catenin is targeted for degradation by the Adenomatous Polyposis Coli (APC)/Axin/Glycogen Synthase Kinase 3 Beta (GSK3β) complex. Radiation suppresses Wnt ligands, accelerating β-catenin degradation and reducing stem-cell survival. When Wnt or R-spondin-1 (RSPO1) is supplied, β-catenin accumulates, translocates to the nucleus, and activates T-cell Factor/Lymphoid Enhancer Factor (TCF/LEF) transcription of stemness genes (e.g., Leucine-rich repeat-containing G-protein coupled receptor 5 (Lgr5), SRY-Box Transcription Factor 9 (Sox9)). Canonical Wnt signaling is essential for intestinal stem cell (ISC) survival and regeneration following irradiation. The RSPO1 enhances β-catenin activity, attenuates radiation-induced gastrointestinal syndrome, and improves survival without conferring tumor protection. Positive feedback regulators, such as Cluster of Differentiation 44 (CD44), can further amplify Wnt activity during epithelial repair, whereas Wnt inhibition impairs crypt restitution [31]. Beyond the intestine, Wnt signaling contributes to fibrotic remodeling and EMT in lung and skin injury, while its crosstalk with TGF-β and Hedgehog pathways coordinates epithelial–stromal communication during recovery [32].

##### Hedgehog-Wingless-related integration site (Wnt) crosstalk

Hedgehog signaling pathway mainly plays a role in promoting the repair of radiation injury. Following intestinal irradiation, damaged intestinal epithelium secretes Sonic Hedgehog (SHH), which binds Patched receptors on mesenchymal cells, activating Glioma-associated oncogene (Gli) transcription factors. These mesenchymal cells, in turn, produce Wnt ligands, creating an epithelial-stromal-epithelial feedback loop that restores stem-cell pools and promotes crypt regeneration. Genetic and organoid studies demonstrate that Desert Hedgehog and Wnt Family Member 2B (WNT2B) are also required for effective post-irradiation repair. In radiation-induced esophagitis (RIE), impaired Hedgehog signaling delays repair, prolonging mucosal ulceration [33].

##### Hippo Pathway

Hippo signaling regulates epithelial plasticity in response to injury. Injury-induced cytoskeletal remodeling suppresses Hippo kinases Macrophage Stimulating 1/2 (MST1/2) and Large Tumor Suppressor Kinase 1/2 (LATS1/2), allowing unphosphorylated Yes-Associated Protein/Transcriptional Coactivator with PDZ-binding Motif (YAP/TAZ) to translocate to the nucleus and bind TEA Domain transcription factor (TEAD). This promotes proliferation, survival, and metabolic reprogramming of epithelial progenitors. In the intestine, YAP/TAZ functions in concert with Wnt, Notch, and prostaglandin pathways, accelerating crypt regeneration. Loss of YAP impairs regenerative capacity, whereas sustained or excessive activation drives dysplastic changes. Thus, precise regulation of YAP/TAZ activity determines whether the intestinal epithelium undergoes controlled regeneration and restoration of normal architecture, or persistent signaling that disrupts homeostasis and fosters dysplastic or pathological changes in irradiated mucosa. In the skin, YAP/TAZ enhances basal keratinocyte proliferation, improving epidermal recovery following radiation burns [34, 35].

##### Nuclear Factor Kappa-Light-Chain-Enhancer of Activated B Cells/p53 Upregulated Modulator of Apoptosis (NF-κB/PUMA) balance

NF-κB acts as a double-edged regulator in radiation injury. In the intestinal epithelium, NF-κB activation via Inhibitor of Nuclear Factor Kappa B Kinase Subunit Beta (IKKβ) promotes transcription of anti-apoptotic genes such as B-cell Lymphoma 2 (Bcl-2) and X-linked Inhibitor of Apoptosis (XIAP), and suppression of p53-PUMA signaling, limiting epithelial apoptosis and maintaining barrier integrity. However, radiation-induced p53 upregulates PUMA, and excessive PUMA expression results in crypt cell loss, leading to mucosal denudation and severe diarrhea. In the brain and skin, NF-ΚB-driven cytokine responses contribute to neuroinflammation and RD, but also orchestrate antioxidant and reparative pathways [36, 37]. In summary, the ultimate effect primarily depends on the surrounding cellular environment and the timing of the activation of this signaling pathway; the relative balance between NF-κB-mediated survival and PUMA-driven apoptosis dictates tissue outcome.

##### Epidermal Growth Factor/ErbB (EGF/ErbB) Pathway

Epidermal Growth Factor Receptor (EGFR) signaling enhances cellular survival after irradiation by facilitating DNA double-strand break repair. Upon radiation exposure, EGFR translocates to the nucleus, associates with DNA-PKcs, and promotes non-homologous end-joining. In normal epithelia such as the gastrointestinal tract and skin, ligand-dependent EGFR activation can promote acute wound healing; however, this benefit is manifested as radioresistance in tumors. This illustrates the tissue- and context-specific trade-offs of EGFR signaling [38, 39].

#### Stress-response, cytoprotection, and DNA-damage signaling

##### Phosphoinositide 3-Kinase-Protein Kinase B/Mechanistic Target of Rapamycin (PI3K-AKT/mTOR) Pathway

The PI3K-AKT pathway integrates trophic and stress signals following irradiation. Radiation-activated growth factors, such as EGF and IGF-1, stimulate PI3K to generate Phosphatidylinositol (3,4,5)-Trisphosphate (PIP3), recruiting and activating AKT. AKT phosphorylates downstream targets, including mTORC1, promoting protein synthesis, DNA repair, and anti-apoptotic signaling. In the intestine, Insulin-like Growth Factor 1 (IGF-1) and basic Fibroblast Growth Factor (bFGF) activate the PI3K-AKT pathway to suppress p53-PUMA-mediated apoptosis, thereby preserving stem cells and reducing acute mucosal injury. However, sustained PI3K-AKT-mTOR activation, accompanied by impaired autophagy, contributes to chronic intestinal damage. In hematopoietic stem cells, AKT activation enhances survival post-irradiation; however, chronic activation impairs autophagy and accelerates mesenchymal stem-cell exhaustion, contributing to long-term marrow suppression. In the brain, astrocytic PI3K-AKT activation inhibits autophagy and promotes excessive Vascular Endothelial Growth Factor (VEGF) secretion, leading to blood–brain barrier (BBB) disruption. Thus, PI3K-AKT functions as an acute pro-repair mediator but drives late toxicity when persistently activated [40].

##### Mitogen-Activated Protein Kinase (MAPK) Pathways

IR activates MAPK cascades that govern early inflammatory and stress responses. Radiation activates Extracellular Signal-Regulated Kinase (ERK), JNK, and p38 pathways. ERK promotes transient proliferation and repair, whereas sustained JNK and p38 activity induce pro-apoptotic and inflammatory genes such as Fas Ligand (FasL) and TNF-α. In the irradiated brain, microglia display Mitogen-Activated Protein Kinase Kinase (MEK)-ERK-c-Jun activation, which amplifies pro-inflammatory gene expression and drives neuroinflammation. Persistent p38 activation correlates with white matter degeneration and cognitive decline after cranial irradiation. In the skin, JNK-driven keratinocyte apoptosis contributes to acute desquamation and ulceration. In peripheral tissues, such as the intestine, MAPK signaling intersects with redox balance and cytoprotective mechanisms, thereby modulating the balance between repair and fibrosis. In the lung, sustained MAPK activation further cooperates with TGF-β to reinforce EMT and promote fibroblast activation [41, 42].

##### Nuclear Factor Erythroid 2-Related Factor 2 (Nrf2) Pathways

The Kelch-like ECH-associated protein 1 (Keap1)/Nrf2 pathway has been demonstrated to exert a protective effect against radiation-induced damage, which can counteract irradiation-induced oxidative stress, enhance DNA damage responses, and restrain chronic inflammation. Radiation-induced ROS oxidize cysteine residues on Keap1, releasing Nrf2 for nuclear translocation. Nrf2 binds antioxidant response elements and upregulates cytoprotective genes, including Heme Oxygenase 1 (HO-1), NAD(P)H Quinone Dehydrogenase 1 (NQO1), and Glutamate-Cysteine Ligase Catalytic Subunit (GCLC). In the oral mucosa, Nrf2 deficiency exacerbates radiation-induced mucositis, whereas pharmacologic activation with dimethyl fumarate reduces epithelial ulceration and accelerates healing. In the lung, Nrf2 overexpression or pharmacologic activation attenuates radiation-induced skin injury and alleviates pulmonary inflammation and fibrosis, whereas Nrf2 deficiency exacerbates tissue radiosensitivity. Therapeutic strategies aimed at activating Nrf2, such as small molecules that disrupt Keap1 binding, are under investigation for RD and RILI [43]. However, precise control of timing and dosage is essential to minimize the risk of radioresistance [43–45].

Understanding the molecular mechanisms of radiation-induced injury is essential for uncovering the fundamental processes that contribute to tissue damage and dysfunction. These molecular alterations, including DNA damage, oxidative stress, and epigenetic modifications, set the stage for more complex cellular responses. At the cellular level, IR exposure triggers a cascade of events, involving the activation of repair pathways, immune responses, and changes in cellular phenotype. These cellular mechanisms drive organ-specific pathology, leading to both acute and chronic effects of radiation-induced injury. In the next section, we turn to a detailed examination of the cellular responses and functional alterations in key tissues, illustrating how molecular damage leads to functional impairments and long-term health consequences.

## Biological effects of ionizing radiation

### Cellular responses

IR provokes a broad spectrum of cellular responses that collectively determine survival, regulated death pathways, and long-term tissue dysfunction.

Apoptosis is triggered by radiation-induced DNA DSBs and oxidative stress, activating the p53-BAX/BAK signaling pathway, which leads to the release of cytochrome C and the activation of caspases-9 and −3. This process removes damaged cells but also causes alveolar epithelial loss in RILI, depletion of intestinal crypt cells in radiation enteritis (RE), and neuronal damage in RIBI [46]. Persistent DNA damage, on the other hand, induces cellular senescence through the upregulation of p16INK4a and p21CIP1, causing growth arrest. Senescent cells secrete pro-inflammatory cytokines and growth factors as part of the SASP, promoting chronic inflammation and fibrosis. For example, senescent type II alveolar cells contribute to pulmonary fibrosis, while osteoblast senescence plays a key role in radiation-induced osteoporosis [47]. Autophagy typically helps maintain cellular survival by degrading dysfunctional mitochondria and regulating ROS levels, but when dysregulated, it can worsen injury. In the intestines, autophagy suppression hinders regenerative processes, while impaired autophagic flux in astrocytes affects BBB integrity after cranial irradiation [47]. IR also activates the NLRP3 inflammasome, leading to pyroptosis via caspase-1-mediated cleavage of gasdermin D. This inflammatory form of cell death disrupts osteoblast and macrophage function in bone, damages intestinal epithelium, and maintains chronic microglial activation in the CNS, resulting in persistent inflammation [48]. If apoptosis is blocked, necroptosis is triggered through Receptor-Interacting Serine/Threonine-Protein Kinase 1/Receptor-Interacting Serine/Threonine-Protein Kinase 3 (RIPK1/RIPK3)-mediated phosphorylation of Mixed Lineage Kinase Domain-Like Pseudokinase (MLKL), causing membrane rupture and the release of damage-associated molecular patterns (DAMPs) [49]. Necroptosis contributes to inflammation and tissue remodeling in the skin, accelerates mucosal injury, hinders epithelial regeneration in the gastrointestinal tract, worsens lung inflammation, and increases tissue damage and fibrosis [50]. Additionally, IR promotes lipid peroxidation and iron-dependent ROS production, leading to the inactivation of Glutathione Peroxidase 4 (GPX4) and suppression of Solute Carrier Family 7 Member 11 (SLC7A11), which triggers ferroptosis. This mechanism has been linked to cognitive dysfunction in RIBI and endothelial damage and gut barrier disruption in RE [51, 52]. Excessive activation of PARP1 following severe DNA breaks depletes Nicotinamide Adenine Dinucleotide (NAD⁺)/ATP. It promotes the release of mitochondrial apoptosis-inducing factor (AIF), leading to widespread DNA fragmentation, known as Parthanatos [53]. This further exacerbates neuronal and intestinal epithelial loss [54]. Finally, inadequate DNA repair and spindle defects lead to mitotic catastrophe, forming multinucleated or giant cells that eventually undergo apoptosis, necrosis, or irreversible cell cycle arrest, leading to the loss of epithelial and crypt cells in rapidly proliferating tissues like the gastrointestinal tract and skin, thus amplifying radiation-induced damage [48].

### Systemic and organ-specific effects

#### Impact factors

Radiation exposure, whether localized or whole-body, induces complex tissue damage, with several common risk factors affecting the severity of radiation-induced injuries across various organs. One of the most significant risk factors is the radiation dose, with higher doses leading to more severe damage. Whole-body irradiation typically results in more widespread and severe damage compared to localized radiation exposure, as it impacts multiple organ systems, especially the hematopoietic and immune systems. Additionally, age is a major determinant of susceptibility, with both pediatric and elderly populations being more vulnerable to radiation-induced tissue damage due to differences in cellular repair capacity and organ function. Gender also plays a role in radiation sensitivity, with females exhibiting greater susceptibility [55, 56]. Concurrent chemotherapy or immunotherapy compounds the effects of radiation, as cytotoxic agents can exacerbate tissue damage and impair tissue repair mechanisms, leading to more pronounced organ dysfunction. The use of anti-angiogenic drug compounds causes radiation damage to mucosal tissues and the vascular endothelium, leading to the chronicity of injury [57]. Furthermore, the basic condition of the organ is an important factor that determines whether radiation damage occurs to the organ; pre-existing comorbidities such as cardiovascular disease, diabetes, and chronic respiratory conditions increase the risk of severe radiation-induced injury [58, 59]. For example, smoking and pre-existing lung disease are recognized as significant risk factors for RILI [60], while vascular comorbidities and hypertension heighten the risk for RIHD [61]. Skin thickness, obesity, and pre-existing skin conditions like psoriasis or eczema make the skin more vulnerable to RD [62, 63]. Graft-versus-host disease (GVHD) or infections can worsen the severity of radiation-induced hematopoietic injury [64].

#### Inflammatory and immune responses.

IR triggers a highly dynamic cascade of immune and inflammatory events, beginning within minutes after exposure and extending into chronic phases. Damaged epithelial and endothelial cells release DAMPs, including HMGB1, ATP, and mitochondrial DNA, which are recognized by pattern recognition receptors (PRRs) such as Toll-Like Receptors (TLRs) and the cGAS-STING pathway. This initiates rapid activation of NF-κB and MAPK signaling, resulting in the production of pro-inflammatory cytokines (e.g., TNF-α, IL-1β, IL-6) and chemokines (e.g., C-X-C Motif Chemokine Ligand 8 (CXCL8), C–C Motif Chemokine Ligand 2 (CCL2)), which recruit immune cells to irradiated tissues [65].

##### Innate immune responses

Neutrophils are among the earliest responders, migrating into damaged tissue and releasing ROS, proteases, and neutrophil extracellular traps (NETs) that exacerbate DNA and membrane damage. Macrophages are subsequently recruited and exhibit functional plasticity. Initially, they polarize toward an M1 phenotype, secreting TNF-α, IL-12, and reactive nitrogen species that sustain inflammation and contribute to tissue injury. With time, macrophages can shift toward an M2 phenotype, producing TGF-β and PDGF that promote fibroblast activation and ECM deposition, driving late fibrosis. Dendritic cells undergo activation via DAMPs and type I interferons, linking innate sensing to adaptive immunity, although excessive activation may aggravate tissue autoimmunity [66, 67].

##### Adaptive immune responses

Lymphocytes are highly radiosensitive; widespread apoptosis of CD4⁺ and CD8⁺ T cells leads to acute immunosuppression and increased infection risk. However, the surviving T-cell pool often displays phenotypic skewing. Th1/Th17 polarization and excessive Interferon Gamma/Interleukin-17 (IFN-γ/IL-17) production amplify chronic inflammation in irradiated organs, while Regulatory T Cell (Treg) expansion may dampen effective tissue repair. B-cell depletion impairs humoral immunity, but surviving B cells may contribute to autoantibody generation in chronic injury contexts. Radiation also enhances antigen presentation and cross-priming, thereby potentiating cytotoxic T-cell responses against irradiated tumor cells.

##### Endothelial and stromal involvement

Endothelial cells exposed to IR upregulate adhesion molecules (Intercellular Adhesion Molecule 1 (ICAM-1), Vascular Cell Adhesion Molecule 1 (VCAM-1), E-selectin), enhancing leukocyte recruitment. Persistent endothelial injury also contributes to chronic hypoxia, perpetuating NF-κB activation and vascular remodeling. Fibroblasts respond to macrophage- and lymphocyte-derived signals by differentiating into myofibroblasts, producing collagen and matrix proteins that culminate in fibrosis [68].

At the organismal level, these local immune events translate into systemic immune dysregulation. Acute radiation syndrome (ARS) is characterized by profound lymphopenia, bone marrow suppression, and cytokine storm-like responses. In sublethal exposures, chronic low-grade inflammation persists across organs, with elevated circulating IL-6 and TNF-α contributing to multi-organ fibrosis and neurovascular injury [69].

#### Radiation-induced hematopoietic injury

IR also causes extensive systemic effects, particularly on the hematopoietic system, which is highly sensitive to radiation-induced damage. High-dose radiation, particularly doses exceeding 2 Gy, significantly increases the risk of hematopoietic suppression.

Hematopoietic stem cells (HSCs) in the bone marrow, responsible for the production of blood cells, are among the first targets. In cases of high-dose radiation, the radiation-induced depletion of HSCs leads to profound hematopoietic suppression, with a marked reduction in the number of circulating leukocytes, erythrocytes, and platelets. This results in conditions such as leukopenia, anemia, and thrombocytopenia, which increase the risk of infection, bleeding, and anemia [64].

The initial response to radiation-induced hematopoietic injury also involves an inflammatory cascade, driven by cytokine release from damaged cells and immune cells. The activation of the NF-κB and MAPK pathways in hematopoietic cells and endothelial cells triggers the release of pro-inflammatory cytokines. These cytokines promote leukocyte recruitment and further exacerbate tissue damage. This creates a feedback loop of inflammation and immune dysregulation. Clinically, patients experience a range of acute symptoms, including fever, malaise, and fatigue, as well as a heightened risk of infections due to immunosuppression.

As the acute inflammatory response progresses, the bone marrow microenvironment undergoes remodeling. The regeneration of HSCs is impaired further, and fibrosis in the bone marrow is promoted. Inflammatory signals also activate macrophages, which contribute to tissue remodeling and further fibrosis. Long-term effects of radiation on the hematopoietic system and immune response include persistent anemia, neutropenia, and thrombocytopenia, which can lead to chronic infections, autoimmune disorders, and an increased risk of secondary cancers [70].

Although there is no completely clear diagnostic method, there are still many diagnostic methods that can assist in the diagnosis of radiation-induced hematological injury. Blood tests, particularly complete blood counts (CBC), are commonly used to monitor changes in white blood cell, red blood cell, and platelet levels, which serve as critical indicators of bone marrow function [71]. In the case of significant radiation exposure, abnormal blood counts can be used to assess the severity of ARS. Bone marrow examination, including biopsy and aspiration, allows for direct evaluation of marrow cellularity, morphology, and any abnormal patterns that may indicate suppression or dysplasia. Imaging techniques such as Magnetic Resonance Imaging (MRI) are increasingly utilized, especially in pediatric populations, as a non-invasive method to assess marrow space alterations. Moreover, molecular biology assays are employed to identify genetic markers associated with radiation-induced damage, including markers of apoptosis and DNA repair mechanisms [72]. Clinical assessment systems, such as the H-module application, can be used to predict the severity of ARS based on hematological parameters, providing valuable insights for clinical decision-making [71].

#### Radiation-induced lung injury (RILI)

In contemporary medical practice, about 5–20% of patients develop RILI within 2–3 months after undergoing radiotherapy. The development of RILI is strongly correlated with the radiation dose, treatment volume, and pre-existing lung function. RILI typically occurs after doses ≥ 30 Gy [73]. Presently, a widely accepted two-stage classification system distinguishes RILI into an early stage (1–3 months post-radiotherapy) known as Radiation-Induced Pneumonitis (RIP) and a late stage (beyond 3 months) referred to as RIPF [74].

Alveolar epithelial cells (AECs) are the most impacted cells in the lung after IR. The primary targets are type I alveolar epithelial cells (AEC I), which make up approximately 90% of the epithelial lining, leading to their apoptosis. Type II alveolar epithelial cells (AEC II), which are responsible for surfactant production and epithelial repair, are also damaged, resulting in a reduction in surfactant secretion. A cascade of inflammatory responses is triggered, with macrophages and neutrophils releasing pro-inflammatory cytokines, perpetuating the inflammatory cycle, and attracting additional immune cells [75].

For example, M1 macrophages further intensify the inflammation by Matrix Metalloproteinases (MMPs) and pro-fibrotic factors such as TGF-β. ROS generated within the lung also exacerbate this inflammatory response, enhancing cytokine production and immune cell activation. In the early stage, pathological changes include the detachment of endothelial and epithelial cells, surfactant loss, and alveolar collapse. Small blood vessel dysfunction, fibrin-rich exudates in the alveoli, and the formation of hyaline membranes are also common. Clinically, patients present with symptoms such as low-grade fever, dry cough, and chest tightness [76].

**Fig. 2:**
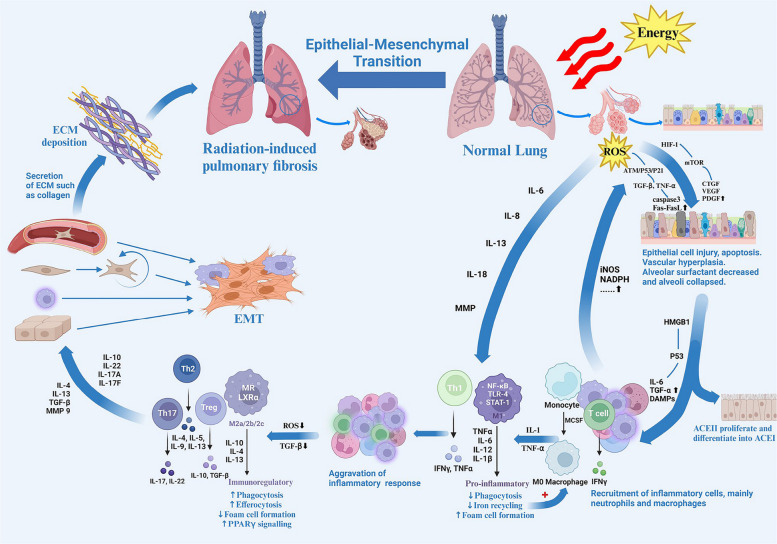
Comprehensive Overview of the Onset and Progression of RILI. Legends: Radiation directly or indirectly (via reactive oxygen species, ROS) damages alveolar epithelial and endothelial cells, triggering local inflammation and immune cell infiltration. Inflammatory mediators and oxidative stress activate fibrotic pathways, leading to fibroblast proliferation and collagen deposition. Chronic fibrosis disrupts normal lung tissue, causing alveolar septal thickening, reduced lung compliance, and ultimately resulting in pulmonary fibrosis

Over time, fibroblasts are recruited to the site of injury, where they proliferate and secrete ECM components, contributing to the development of pulmonary fibrosis. The EMT in AEC II cells is a key process in the progression of RIPF, which promotes the accumulation of myofibroblasts [77]. In the later stage, pathological findings include the proliferation of lung epithelial cells and myofibroblasts, collagen deposition in the interstitial spaces, and the widening of alveolar spaces. These changes result in a further reduction in lung compliance and capacity. Symptoms progress to worsening dyspnea, persistent dry and choking cough, and, in severe cases, can lead to chronic respiratory failure (Fig. 2) [78, 79].

Some diagnostic methods can assist in the diagnosis of RILI. Chest Computed Tomography (CT) is the primary tool for detecting early signs like ground-glass opacities and consolidation. Chest X-rays show less sensitivity [80]. Bronchoscopy with bronchoalveolar lavage fluid (BAL) helps rule out infections and malignancies. Pulmonary function tests assess restrictive capacity. Emerging techniques such as diffusion weighted imaging (DWI), Single-Photon Emission Computed Tomography/Computed Tomography (SPECT/CT), and Positron Emission Tomography (PET) scans with Fluorodeoxyglucose F18 (18F-FDG) offer enhanced diagnostic accuracy [81, 82].

#### Radiation Enteritis (RE)

RE, also called radiation-induced intestinal injury (RIII), is a functional disorder of the large and small bowel that mainly occurs during or after a course of radiotherapy. According to time and pathological features, RE can be divided into Acute Radiation Enteritis (ARE) in the early stage and chronic RE (CRE), which occurs after 6 months of treatment. Despite advances in technology, 40% of patients can be accompanied by significant lesions at the radiation dosage of 10–30 Gy, and the number will sharply increase to 90% with the dosage beyond 30 Gy [83].

Due to the high regenerative capacity, intestinal stem cells in the crypts are particularly vulnerable to IR. Low doses of radiation induce cell cycle arrest, impairing the regenerative potential of these stem cells, while high doses lead to the complete loss of their regenerative capacity. As radiation damage progresses, cytochrome C is released, triggering caspase activation and apoptosis in the affected cells. A loss of epithelial cells leads to decreased villus height and intestinal barrier dysfunction, which increases susceptibility to infection and inflammation, resulting in ARE [84]. Clinically, patients experience acute symptoms, including nausea, abdominal pain, diarrhea, and loss of appetite.

**Fig. 3:**
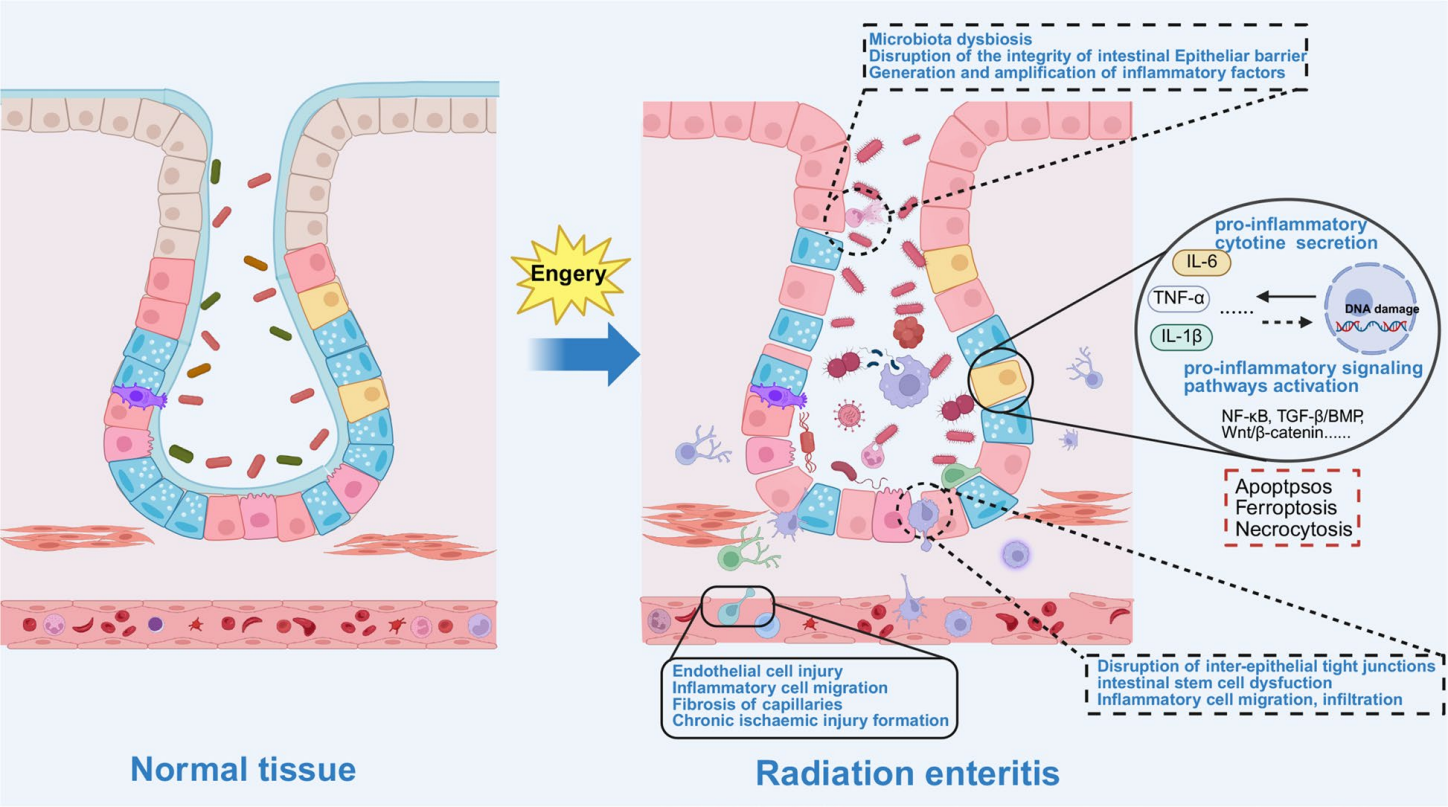
Potential changes during the process of radiation enteritis. Legends: Radiation directly or indirectly (via reactive oxygen species, ROS) triggers apoptosis in intestinal epithelial and endothelial cells, causing inflammation and disruption of the intestinal epithelial barrier, further leading to gut microbiota dysbiosis, exacerbating the inflammatory response, and resulting in edema and hemorrhage. Persistent inflammation ultimately leads to mucosal atrophy, fibrosis, and intestinal dysfunction

The destruction of the vasculature within the intestinal wall further exacerbates the injury. Radiation-induced damage to vascular endothelial cells increases vascular permeability, leading to inflammation and structural changes in the endothelium. Over time, this contributes to fibrosis of the capillaries supporting the intestine, a hallmark of CRE [85]. IR also disrupts the intestinal microbiota, leading to dysbiosis. There is a reduction in beneficial bacteria, including Bifidobacterium, Faecalibacterium prausnitzi, and Clostridium cluster XIVa, and an increase in pathogenic bacteria, such as Enterobacteriaceae and Bacteroidetes. These pathogenic microbes inhibit the growth of probiotics and activate the NF-κB pathway, further escalating local inflammation and damaging the intestinal barrier. Additionally, radiation toxicity can also cause microbiota translocation and endotoxin release, triggering systemic inflammation (Fig. 3) [86]. Approximately 5%−10% of patients develop chronic symptoms such as weight loss, anorexia, persistent diarrhea, and infections, particularly following pelvic radiotherapy. In severe cases, radiation-induced injury may lead to intestinal necrosis, bowel perforation, and even death [87, 88].

Several diagnostic methods can assist in the diagnosis of RE. Symptom assessment is supported by endoscopy with biopsy as the gold standard. CT and MRI reveal mural thickening, fistulas, or strictures, with diffusion-weighted MRI providing added sensitivity. Capsule endoscopy and double-balloon enteroscopy expand the evaluation of small bowel involvement. Functional tools include citrulline levels and fecal calprotectin; radiomics and machine learning applied to CT/MRI are emerging to stratify risk and severity [89–91].

#### Radiation-Induced Brain Injury (RIBI)

Approximately 10–20% of patients who undergo radiotherapy for tumors, especially head and neck, experience RIBI. And it is typically observed after doses ≥ 30 Gy [92, 93].

BBB is a critical structure that protects the brain from harmful substances by selectively regulating the passage of molecules. However, radiation exposure to the CNS leads to significant damage in the endothelial cells constituting the BBB. This damage increases BBB permeability and disrupts its selective barrier function, facilitating the entry of inflammatory mediators and harmful substances into the brain parenchyma, which initiates neuroinflammation [94].

Microglia become activated and transition into the M1 pro-inflammatory phenotype. In this state, microglia release a variety of cytokines, including TNF-α, IL-6, and IL-1β, which amplify the inflammatory response and lead to neuronal dysfunction. Astrocytes, which are responsible for maintaining BBB integrity, also respond to radiation by switching between neurotoxic A1 and neuroprotective A2 phenotypes. This polarization, regulated by inflammatory signals, further contributes to neuroinflammation and exacerbates neuronal damage. Due to the BBB disruption, white-matter injury, and cerebral oedema, the typical clinical symptoms of acute RIBI include headache, nausea, vomiting, and lethargy [95–97].

**Fig. 4:**
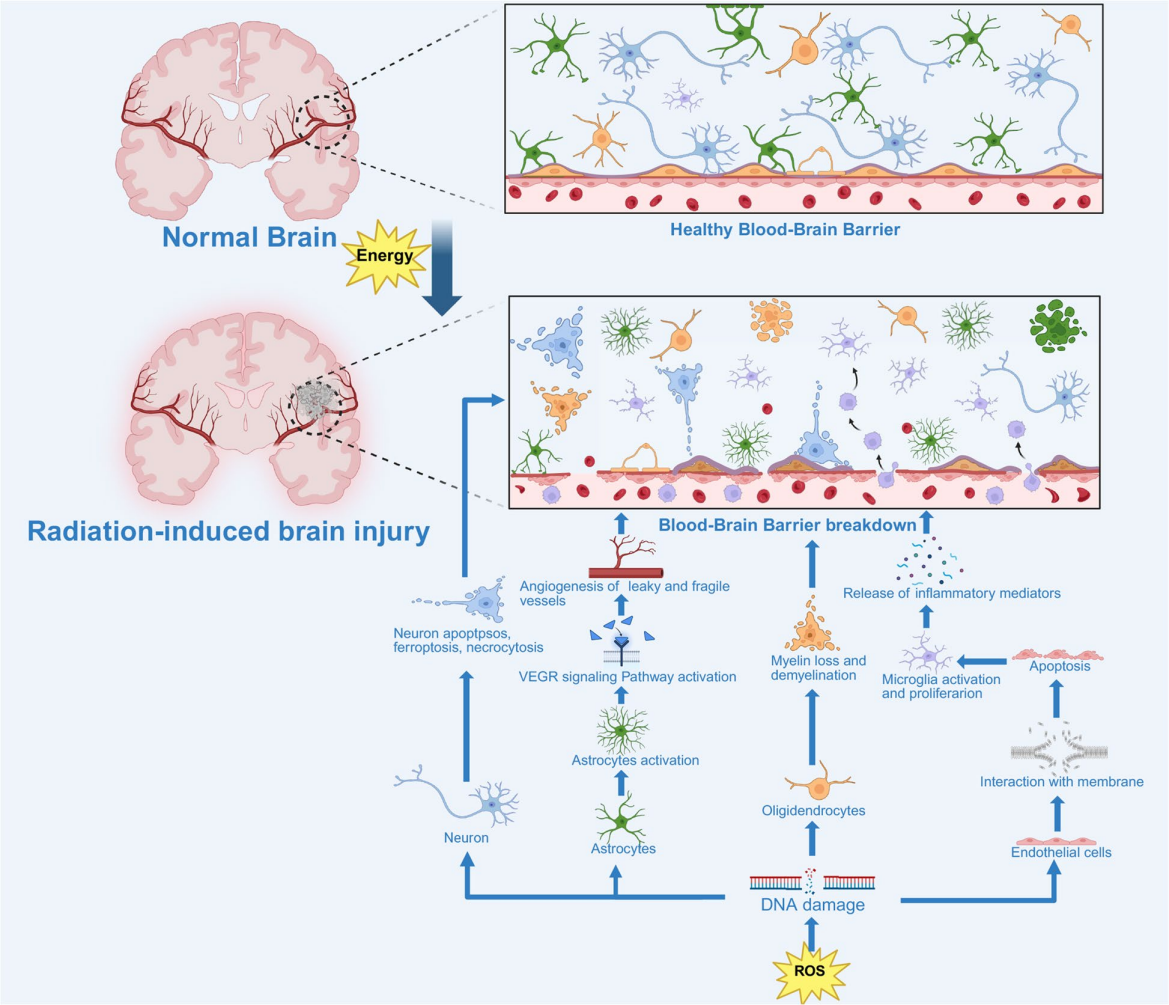
Potential changes during the process of radiation-induced brain injury. Legends: High-energy radiation induces oxidative stress and inflammatory responses, damaging neurons and glial cells. The release of inflammatory mediators such as cytokines and chemokines further exacerbates brain injury, resulting in blood–brain barrier disruption, local neuroinflammation, and edema. Then, the reduction in cerebral blood flow and white matter degeneration gradually occurs. Ultimately, sustained tissue damage triggers neurological dysfunction

Additionally, IR exposure can activate various signaling pathways such as PI3K/AKT and VEGF, which promote further endothelial damage and disrupt the repair processes that are necessary for BBB recovery. This exacerbates the loss of BBB function and leads to ongoing neuroinflammatory responses (Fig. 4). And due to the irreparable necrosis of neurons and reduced neurogenesis, for chronic RIBI, the typical clinical symptoms are cognitive deficits, memory loss, mood changes, seizures, and so on [40, 97].

Fluid attenuated inversion recovery (FLAIR) and contrast enhancement show radiation-induced edema and necrosis. Advanced sequences improve specificity, DWI differentiates necrosis from recurrence, perfusion MRI assesses relative cerebral blood volume (rCBV), and MR spectroscopy detects lipid–lactate elevation. PET with amino acid tracers (O-(2-18F-fluoroethyl)-L-tyrosine (FET), L- [11C]methionine (MET)) offers metabolic distinction. More recently, hybrid PET/MRI and radiomics-based models provide higher diagnostic accuracy, while liquid biopsy of circulating exosomal miRNAs is under investigation for early detection [98–101].

#### Radiation Dermatitis (RD)

RD, also known as radiodermatitis, radiation-induced skin injury (RISI), is recognized as the most common side effect of radiotherapy. The risk of RD appears after radiotherapy for many kinds of cancers, especially for head and neck cancers, and the risk has reached as high as 100%. According to the occurrence time, RD is divided into acute RD (ARD), occurring approximately 2–3 weeks following the initial irradiation, and CRD, occurring at least 90 days or even years after radiotherapy. Typically, ARD can be observed after doses ≥ 2 Gy [102].

In the acute phase, IR causes the necrosis of keratinocytes, epidermal hyperplasia, hyperkeratosis, and subepidermal edema. In the dermis, vascular dilation, increased capillary permeability, and fibroblast proliferation are observed. Immune cells are recruited to the site of injury and release cytokines that further exacerbate the inflammatory response [103]. These lead to erythema, blistering, desquamation, and pruritus.

**Fig. 5:**
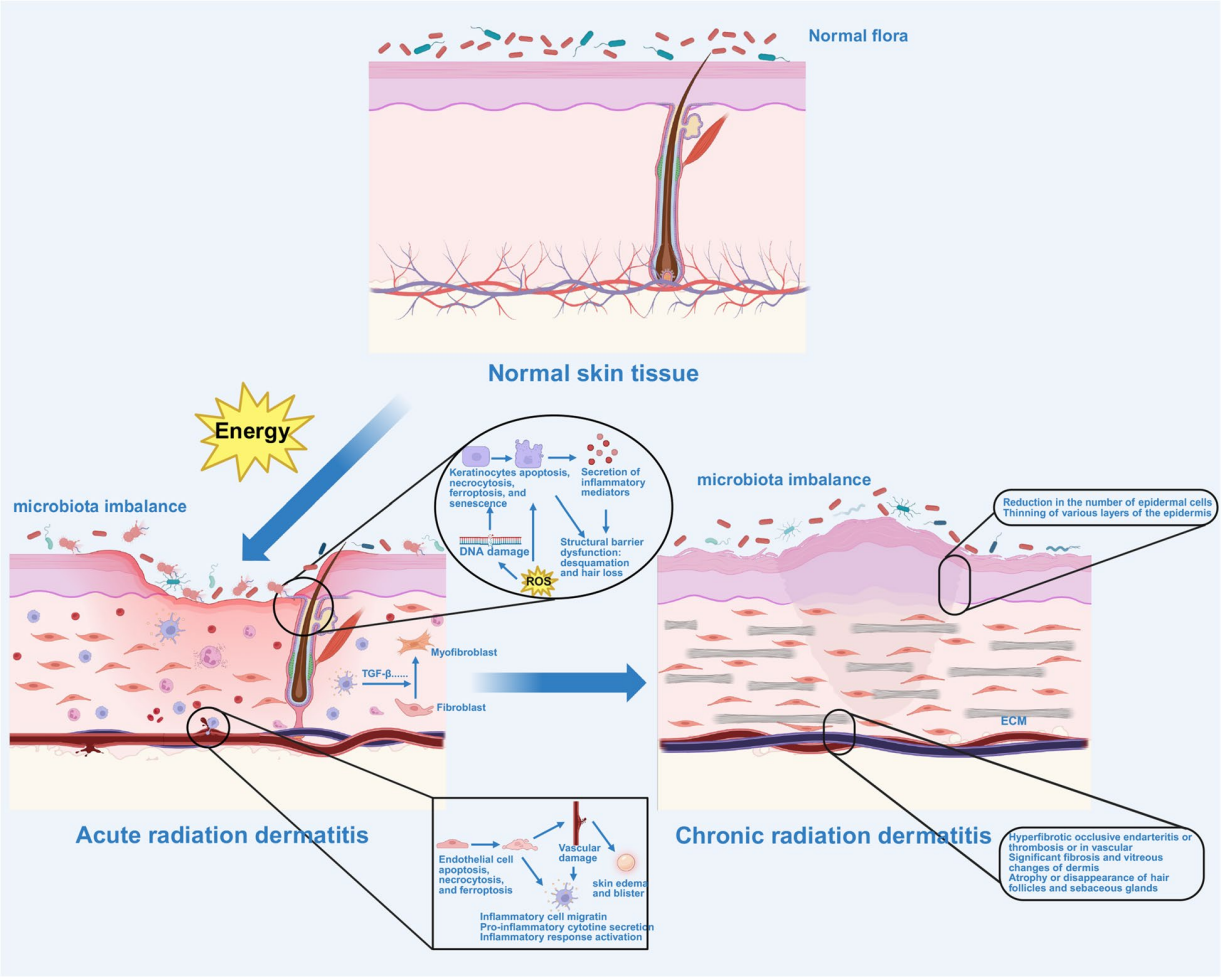
Potential changes during the process of radiation dermatitis.Legends: Radiation exposure damages the stratum corneum, epidermis, and dermal cells, triggering localized inflammation characterized by erythema, edema, and skin dryness. The release of inflammatory mediators increases vascular permeability and immune cell infiltration, compromising the skin barrier. Over time, repeated cycles of cellular damage and repair may lead to skin fibrosis, pigmentation, or ulceration

In the chronic phase, the damage of IR to actively dividing basal keratinocytes, melanocytes, and hair follicle stem cells persists. The skin’s epidermal layer becomes thinner as a result of the loss of these critical stem cells. Fibroblasts in the dermis activate and proliferate, promoting collagen synthesis and resulting in skin sclerosis and decreased elasticity. The appendages, including hair follicles and sebaceous glands, may degenerate (Fig. 5) [104, 105]. Thus, for CRD, typical symptoms are skin atrophy, hyperpigmentation, fibrosis, and ulceration [106]. Notably, CRD can lead to skin cancer, so long-term follow-up is necessary [107].

Clinical scoring systems (Radiation Therapy Oncology Group (RTOG), Common Terminology Criteria for Adverse Events (CTCAE)) remain first-line for diagnosing RD. Histology confirms keratinocyte apoptosis and dermal fibrosis. High-frequency ultrasound quantifies dermal thickness; optical coherence tomography and laser Doppler imaging assess microvascular changes. Infrared thermography monitors inflammation, and multiphoton microscopy provides real-time, noninvasive imaging of collagen remodeling. Artificial intelligence–assisted image analysis is under development to standardize grading [108, 109].

#### Radiation-induced bone injury

Approximately 5–30% of patients receiving radiotherapy to the pelvic region experience radiation-induced bone injury, and it typically occurs after doses ≥ 30 Gy.

**Fig. 6:**
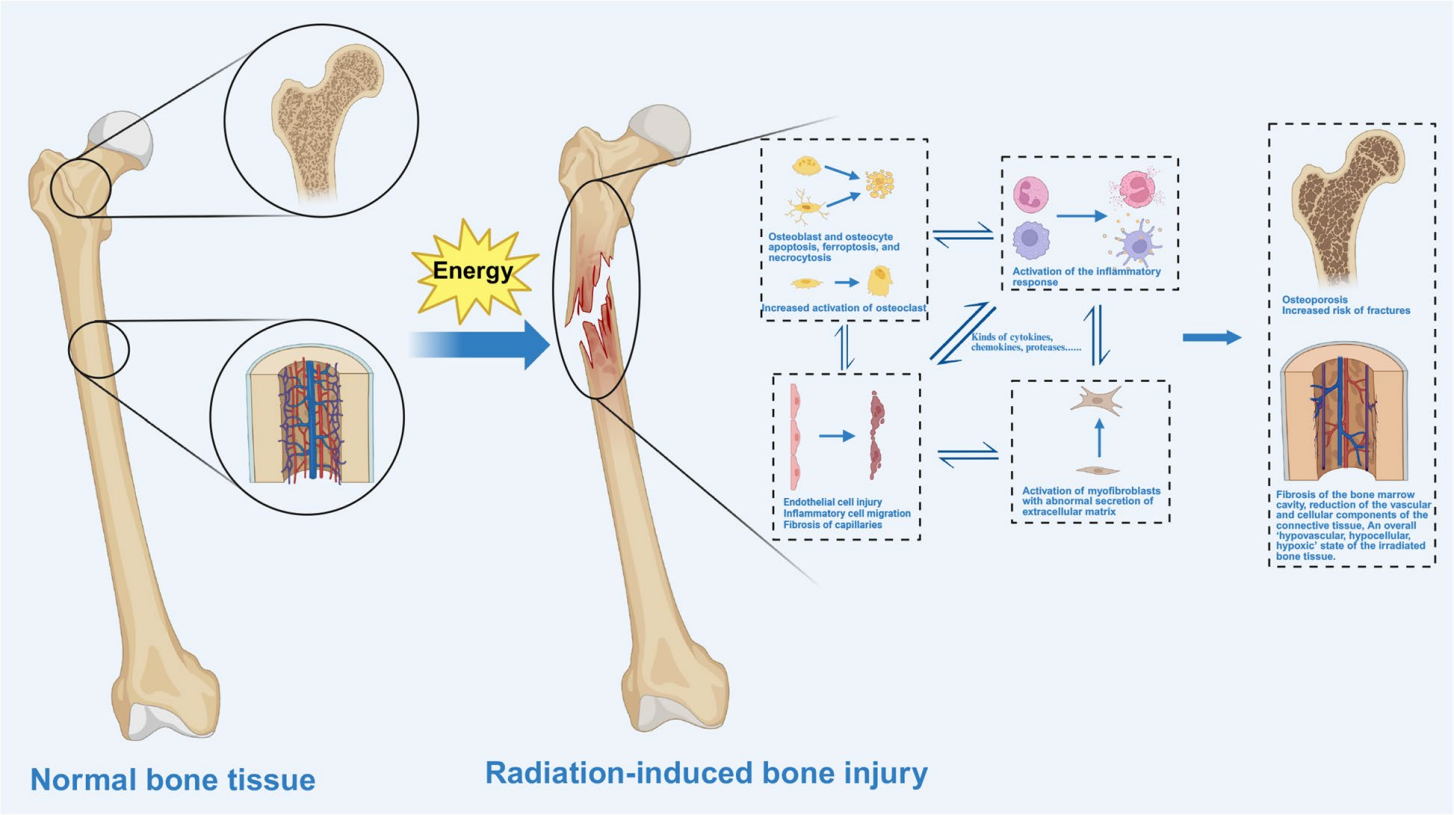
Potential changes during the process of radiation-induced bone injury. Legends: Radiation directly damages bone marrow cells, osteoblasts, and osteocytes, impairing bone remodeling and mineralization. This damage triggers an inflammatory response, with the release of cytokines and growth factors, which activate osteoclast-mediated bone resorption and inhibit osteoblast activity. Over time, the balance between bone formation and resorption is disrupted, leading to reduced bone density, bone marrow fibrosis, and an increased risk of fractures. Chronic radiation exposure can result in permanent alterations in bone structure and function

Bone tissue is sensitive to IR, with osteoblasts and osteoclasts being particularly affected. Osteoblasts undergo apoptosis following radiation exposure, resulting in diminished osteogenic activity and impaired bone matrix synthesis. This attenuation in osteoblast function disrupts the balance between bone formation and resorption, contributing to decreased bone mineral density. Osteoclasts, while initially exhibiting reduced activity post-irradiation, may undergo compensatory hyperactivity over time [110, 111]. This imbalance favors bone resorption, exacerbating bone loss. Furthermore, radiation exposure activates the NLRP3 inflammasome in bone marrow-derived macrophages (BMDMs), leading to pyroptosis (Fig. 6) [112]. Localized pain and swelling are the common clinical symptoms of acute radiation-induced bone injury, and then symptoms of the chronic phase, like fractures, osteonecrosis (ORN), and even bone marrow suppression, will occur [58, 113].

X-ray and CT remain standard for detecting radiation-induced cortical disruption. MRI identifies marrow necrosis and fistula tracts. PET/CT detects early metabolic changes, while dual-energy CT improves sensitivity for cortical lesions. Biopsy distinguishes necrosis from recurrence. Biomarkers such as circulating Nrf2 and bone turnover markers are being explored for early screening [113].

#### Radiation-induced heart disease (RIHD)

In the cardiac tissue, IR primarily injures the coronary endothelium, driving microvascular rarefaction, ROS-mediated inflammation, and pro-thrombotic signaling; cardiac fibroblasts acquire myofibroblast phenotypes with excess collagen deposition, while cardiomyocytes exhibit mitochondrial dysfunction and death, resulting in radiation-induced myocardial fibrosis [114]. Typical RIHD includes conditions such as coronary artery disease, heart failure, cardiomyopathy, pericardial disease, conduction system dysfunction, and valvular disease. Typical symptoms are chest pain, dyspnea, and arrhythmias. The incidence of RIHD varies from 4.1% to 62%, depending on the radiation dose, irradiation field, and pre-existing cardiovascular conditions. It is reported that the risk of major coronary events rises by 7.4% per Gy mean heart dose [115].

Echocardiography is first-line, assessing systolic/diastolic function. Electrocardiogram (ECG) detects arrhythmias. Biomarkers (troponins, N-terminal pro-Brain Natriuretic Peptide (NT-proBNP)) provide early signals of injury. Cardiac MRI offers superior detection of fibrosis and edema, while coronary CT angiography quantifies atherosclerotic burden. Strain imaging and speckle-tracking echocardiography improve sensitivity for subclinical dysfunction. Nuclear techniques such as SPECT and PET assess perfusion deficits [116].

#### Radiation-induced esophagitis (RIE)

The incidence of acute RIE ranges from 5% to 72% among patients receiving chest radiotherapy, depending on the radiation dose and field. In the esophagus, basal epithelial progenitors incur DNA damage and depletion. Due to basal progenitor loss and mucosal inflammation, acute clinical symptoms are odynophagia, dysphagia. Submucosal fibroblasts and cancer-associated fibroblasts (CAFs) resist apoptosis yet adopt senescence and pro-fibrotic programs that remodel ECM and promote myofibroblast differentiation, reinforced by persistent inflammatory signaling. Due to persistent inflammation and fibrotic remodeling, the symptoms of dysphagia and odynophagia are aggravated, the weight of patients is gradually reduced, and few cases, have perforation or fistula [107].

Endoscopy with biopsy remains the gold standard, visualizing ulceration, strictures, and telangiectasia. Barium swallow outlines structural narrowing, while CT and MRI stage transmural involvement. Endoscopic ultrasound improves the assessment of wall layers. More recently, confocal laser endomicroscopy provides in vivo histology, and esophageal impedance-pH monitoring evaluates functional sequelae [117].

#### Radiation-induced gonadal injury

Radiation-induced gonadal injury refers to the dysfunction of the gonads (testes or ovaries) after radiotherapy. In the testes, undifferentiated spermatogonia are selectively ablated, disrupting spermatogenesis; Sertoli cells are relatively radioresistant at lower doses, whereas Leydig cell steroidogenesis declines with higher exposures. In the ovaries, oocytes within primordial follicles are exquisitely radiosensitive, with substantial follicle depletion at 1–2 Gy, accompanied by granulosa-cell apoptosis and stromal remodeling [118]. Moreover, radiation-induced germ-cell loss can lead to the dysfunction of the hypothalamus-pituitary-gonad axis, which leads to hormonal insufficiency further. Due to these direct or indirect damages, the common manifestations of females are ovarian insufficiency, amenorrhea, and infertility. Male patients are likely to become oligo-/azoospermia and hypogonadism [119].

Some diagnostic methods can assist in the diagnosis of radiation-induced gonadal injury. In men, semen analysis and hormonal assays (Follicle-Stimulating Hormone (FSH), LH, testosterone) evaluate function, with scrotal ultrasound for structure. In women, Anti-Müllerian Hormone (AMH) and FSH provide measures of ovarian reserve, supplemented by antral follicle count on transvaginal ultrasound. MRI can assess ovarian volume and perfusion. Novel approaches include microfluidic sperm analysis and serum metabolomics to refine the detection of subfertility [118].

## Advanced molecular and cellular profiling technologies

Recent advancements in high-throughput technologies, such as single-cell RNA sequencing (scRNA-seq), spatial transcriptomics, and radiogenomics, have significantly enhanced our understanding of radiation-induced tissue remodeling. These innovative techniques provide deeper insights into the complex and heterogeneous cellular responses to radiation-induced injury, offering unprecedented resolution of the dynamic processes involved in both acute and chronic radiation effects.

### Single-cell RNA sequencing (scRNA-seq)

scRNA-seq has enabled the characterization of individual cell types within irradiated tissues, unveiling distinct transcriptional profiles and cellular responses that were previously obscured in bulk tissue analysis. This method has been pivotal in mapping cellular heterogeneity in tissues affected by radiation, such as the lung, intestine, and skin. scRNA-seq has revealed radiation-induced changes in gene expression, particularly in pathways related to inflammation, fibrosis, and DNA damage repair, all of which contribute to tissue injury and remodeling. It has also identified radiation-responsive cell populations, including fibroblasts, immune cells, and endothelial cells, thus advancing our understanding of the cellular mechanisms behind radiation-induced damage [120, 121].

### Spatial transcriptomics

Spatial transcriptomics, another breakthrough technology, integrates gene expression data with tissue architecture, allowing for the precise localization of cellular responses within the tissue microenvironment. This approach is particularly valuable in the study of radiation-induced injury, as it reveals how radiation influences the spatial distribution of immune cells, inflammatory responses, and fibroblast activation within affected tissues. For instance, spatial transcriptomics has been employed to map these cellular interactions in organs such as the lung and gastrointestinal tract, providing new insights into the tissue remodeling processes that occur after IR exposure [122].

### Radiogenomics

Moreover, radiogenomics, the integration of genomic data with radiation exposure, has become an essential tool for identifying genetic factors that influence individual susceptibility to radiation-induced damage and long-term sequelae. By analyzing genomic variations alongside radiation response data, radiogenomics helps uncover biomarkers that predict radiation sensitivity, paving the way for personalized treatment strategies. This field shows particular promise in predicting the risk of radiation-induced complications, such as fibrosis, cancer, and cognitive decline, and holds the potential for advancing precision medicine in radiotherapy [123].

Collectively, these technologies have transformed radiation research by providing a comprehensive, multi-dimensional view of the biological effects of radiation at both the molecular and cellular levels. The integration of scRNA-seq, spatial transcriptomics, and radiogenomics not only deepens our understanding of tissue remodeling in response to radiation but also lays the groundwork for the development of more targeted and effective therapeutic interventions [124–126].

The elucidation of molecular and cellular mechanisms, together with the characterization of clinical manifestations and risk factors across organ systems, provides a comprehensive framework for understanding radiation-induced injury. Bridging these findings to clinical practice requires translating pathogenic pathways into targeted interventions. We therefore summarize current therapeutic approaches, ranging from conventional anti-inflammatory and antifibrotic agents to novel modalities such as stem cell therapy, exosome-based interventions, and nanomedicine, with an emphasis on their mechanisms of action, clinical applications, and future potential.

## Therapeutic targets and strategies

### Anti-inflammatory and immunomodulatory strategies

#### Glucocorticoids (GCs)

Glucocorticoids are among the earliest and most widely used agents for radiation-induced injury. Their therapeutic effect is mediated through binding to the glucocorticoid receptor, which translocates to the nucleus and suppresses pro-inflammatory transcription factors such as NF-κB and AP-1 [127]. This reduces expression of cytokines, including TNF-α, IL-1, and IL-6. Glucocorticoids also induce the expression of MAPK phosphatase-1 (MKP-1), which attenuates MAPK signaling cascades, further lowering inflammation and fibroblast activation [128].

Currently, glucocorticoids remain the cornerstone for managing RIPF, where they dampen alveolar inflammatory infiltrates and improve symptoms such as cough, fever, and dyspnea. In the central nervous system, they reduce vasogenic edema associated with radiation necrosis. For skin toxicity, topical or short systemic courses are sometimes used for severe ARD, while in the gastrointestinal tract, glucocorticoids can transiently relieve ARE [104, 129–131]. However, their efficacy decreases in established fibrosis, where inflammation is no longer the dominant process. Long-term use is also limited by risks of infection, metabolic complications, and impaired wound healing [127].

#### Macrolides (Azithromycin)

Azithromycin and related macrolides have immunomodulatory activity independent of their antibacterial effects. They suppress MAPK and NF-κB signaling, reduce chemokines such as IL-10, and lower pro-inflammatory cytokine expression [132]. Macrolides have been explored as adjunctive therapy for RILI, improving symptoms and radiographic resolution [133].

#### Other Immunomodulators

Thalidomide, an immunomodulatory drug. It suppresses the expression of VEGF, reducing the formation of new blood vessels and alleviating tissue edema and inflammatory responses associated with radiation-induced injury. Additionally, thalidomide modulates inflammatory cytokines such as TNF-α, IL-1, and IL-6, thereby mitigating the inflammatory cascade triggered by radiation. Its anti-fibrotic effects are mediated through the regulation of the TGF-β/Smad3 signaling pathway, inhibiting the activation of fibroblasts and the deposition of collagen, which reduces fibrosis in irradiated tissues. Several studies have demonstrated the potential of thalidomide in the treatment of radiation-induced injury. In a Phase II clinical trial, thalidomide was tested for its efficacy in alleviating RIBI. The results showed significant reductions in brain edema and improvements in cognitive function in a subset of patients [134]. In animal studies, thalidomide has also been shown to reduce RIPF [135]. Furthermore, thalidomide has been explored in the management of radiation-induced oral mucositis [136]. In addition to these applications, thalidomide is being investigated in clinical trials for RIE and other forms of radiation-induced injuries. The drug has shown good tolerability, with mild side effects such as drowsiness, rash, and dizziness, but no significant adverse events in most patients.

Memantine, an N-Methyl-D-Aspartate (NMDA) receptor antagonist, reduces excitotoxicity and neuronal death. Large phase III trials demonstrated that hippocampal-avoidance whole brain radiotherapy (HA-WBRT) plus memantine significantly preserved cognitive function compared to conventional WBRT, establishing this approach as a new standard in managing RIBI.

### Antioxidant and redox-modulating strategies

#### Broad-spectrum antioxidants

Amifostine is a pro-drug activated by alkaline phosphatase in normal tissues. It functions as a free radical scavenger, neutralizing ROS generated during irradiation and reducing DNA double-strand breaks. Beyond its antioxidative properties, Amifostine modulates signaling pathways, including TGF-β/Smad2 and PI3K-AKT-mTOR, thereby reducing EMT and subsequent fibrosis [137]. Clinically, Amifostine has been used as a radioprotectant in thoracic radiotherapy to lower the incidence of RILI and RIE. Importantly, trials suggest that Amifostine does not compromise tumor control, making it a viable cytoprotective adjunct. However, its use is limited by acute side effects such as nausea, vomiting, and hypotension [138].

N-Acetylcysteine (NAC) replenishes intracellular glutathione stores, one of the principal cellular antioxidants. It also has direct free radical scavenging activity. By maintaining redox homeostasis, NAC prevents disruption of epithelial tight junctions and preserves mucosal barrier function after radiation [139]. NAC has been evaluated mainly in RE, where it can decrease inflammatory cytokine expression and reduce diarrhea. Some pilot studies suggest benefit in preventing late mucosal injury, though larger controlled trials are needed to validate efficacy [140, 141].

Melatonin, a pineal hormone, exerts strong antioxidant and anti-inflammatory actions. It can directly neutralize free radicals and upregulate antioxidant enzymes such as superoxide dismutase and glutathione peroxidase. Radiation studies demonstrate that melatonin reduces apoptosis markers, attenuates cytokine release, and improves epithelial regeneration [142, 143]. Clinical application has focused on skin, oral mucosa, and gastrointestinal toxicity. Its favorable safety profile makes it an attractive adjunct [144].

#### Enzyme mimetics and specialized antioxidants

Superoxide dismutase (SOD) mimetics, such as avasopasem manganese (GC4419), replicate the activity of endogenous superoxide dismutase, catalyzing the conversion of superoxide radicals into hydrogen peroxide and oxygen. By mitigating the burst of superoxide generated after irradiation, they interrupt the cascade of lipid peroxidation and DNA damage. Topical formulations of SOD have shown promise in alleviating RD, while systemic agents such as avasopasem have reduced the duration and severity of oral mucositis in head and neck cancer chemoradiation. Ongoing studies are expanding these agents into RILI and RE, where oxidative stress is central to pathogenesis.

α-lipoic acid is a dithiol compound capable of redox cycling, serving as a cofactor in mitochondrial enzymatic complexes. It can regenerate other antioxidants, including vitamins C and E, and thus strengthens the antioxidant network. It has been explored for use in neurological radiation toxicity, aiming to protect mitochondrial function in neurons and glial cells. In the gastrointestinal tract, it has been proposed to reduce oxidative stress associated with mucosal injury [145].

#### Other antioxidant compounds

Pentoxifylline (PTX) improves red blood cell deformability and microcirculation, while vitamin E serves as a potent antioxidant. Together, they reduce TNF-α and fibrosis markers. This combination has been confirmed to improve functional and imaging outcomes of RIPF and RD. Some trials have also extended its use to RE and ORN [146].

Other antioxidants, including reduced glutathione (GSH), ambroxol, PM014, resveratrol, and soy isoflavones, Vanillin, and some derivatives of these antioxidants, such as VND3207 (4-hydroxy-3,5-dimethoxybenzaldehyde), have also been shown to inhibit oxidative reactions [147]. These antioxidants reduce the underlying mechanisms of subsequent damaging inflammatory reactions. Preclinical and clinical studies have confirmed the preventive and therapeutic effects of these antioxidants on radiation-induced injuries such as RIPF, RE, and RIE [148–151].

### Antifibrotic and signaling pathway-targeted strategies

#### Renin–Angiotensin–Aldosterone System (RAAS) inhibitors

The renin–angiotensin–aldosterone system (RAAS) contributes to radiation-induced injury by enhancing oxidative stress, promoting fibroblast activation, and stimulating TGF-β signaling. Angiotensin II, through AT1 receptor binding, drives vascular dysfunction and collagen synthesis. Angiotensin-Converting Enzyme (ACE) inhibitors and Angiotensin II Receptor Blockers (ARBs) interrupt this cascade, reducing vascular permeability and fibrogenesis.

In RILI, preclinical studies suggest that patients taking ACEI/ARB for hypertension had lower rates of pneumonitis and fibrosis. In RIBI, RAAS blockade has shown potential to preserve cognitive function post-radiotherapy by protecting hippocampal microvasculature [152, 153].

#### Small molecule pathway inhibitors

Notable TGF-β inhibitors, such as pirfenidone (PFD), SM16, and LY2109761, are employed to suppress TGF-β production and specifically obstruct downstream signaling pathways. It inhibits fibroblast proliferation and collagen synthesis, thereby attenuating fibrotic remodeling [76, 154]. It is established in idiopathic pulmonary fibrosis (IPF) and increasingly applied to RILI, both for prophylaxis and treatment [155]. Multiple trials are underway in lung cancer patients receiving thoracic radiotherapy, as well as in patients with esophageal cancer treated with chemoradiation. While generally well tolerated, gastrointestinal side effects and photosensitivity require monitoring [156].

Nintedanib is a multikinase inhibitor targeting VEGF, platelet-derived growth factor (PDGF), and Fibroblast Growth Factor (FGF) receptors. Blocking these pathways suppresses the recruitment, proliferation, and differentiation of fibroblasts. Nintedanib combined with corticosteroids has improved outcomes in patients with established RIPF, reducing pulmonary exacerbations and progression to fibrosis. This suggests it may serve as a valuable adjunct for managing symptomatic RILI [157].

VEGF inhibitors, such as Bevacizumab, have been confirmed to reduce vascular permeability and abnormal angiogenesis, thereby limiting edema and fibrovascular proliferation. Bevacizumab has become an established option for RIBI, where it produces symptomatic and radiographic improvement superior to corticosteroids [158]. Endostatin has been evaluated for RILI, though data remain preliminary [159].

Statins, beyond lipid-lowering, exert pleiotropic effects including endothelial stabilization, anti-inflammatory activity, and TGF-β suppression. ROCK inhibitors directly target RhoA-ROCK signaling, preventing cytoskeletal contraction, endothelial barrier disruption, and fibroblast activation [160]. Studies show that ROCK inhibition reverses established fibrosis, while statins mitigate vascular dysfunction and inflammation [161]. Clinically, these agents are being evaluated as adjuncts to reduce pulmonary and vascular radiation-induced injury.

D-penicillamine, an inhibitor of collagen synthesis, was proposed as a potential treatment for RIPF. D-penicillamine, which exhibits a pronounced affinity for lung tissue, acts as a heavy metal chelator and can impede the maturation of salt-soluble collagen in vivo. Several randomized studies confirmed that combination penicillamine and Vitamin E therapy reduces fibrosis in the lung and gastrointestinal late effects. Despite its significant efficacy, D-penicillamine's clinical application is hindered by its relatively slow onset of action [162].

Selegiline is a dipeptidyl peptidase IV (DPP4) inhibitor. A study found that selegiline administration significantly reversed the increase in MDA in the intestinal tissues of irradiated mice. Meanwhile, selegiline treatment further promoted the activation of the Nrf2 signaling pathway in irradiated intestinal tissues and HIEC-6 cells and reduced RE in mice [163].

#### Biologics and monoclonal antibodies

Connective tissue growth factor (CTGF) acts downstream of TGF-β to promote fibroblast proliferation and ECM deposition. Pamrevlumab, a monoclonal antibody against CTGF, interrupts this pathway. While its main development is in IPF, the mechanistic rationale strongly supports its use for RIPF, and translational work is ongoing to bridge this evidence into the radiation-induced injury domain [164].

Checkpoint inhibitors (Programmed Cell Death Protein 1/Programmed Death-Ligand 1 (PD-1/PD-L1), Cytotoxic T-Lymphocyte-Associated Protein 4 (CTLA-4)) are already well established in oncology, but their role in radiation-induced injury is dual. On one hand, excessive checkpoint blockade may exacerbate tissue inflammation; on the other hand, carefully timed modulation can enhance the resolution of fibrosis and promote adaptive repair. Preclinical models show that targeting IL-6 signaling or reprogramming macrophages from a pro-fibrotic M2 phenotype to a reparative phenotype reduces pulmonary fibrosis after thoracic irradiation. Similarly, interventions aimed at modulating Tregs and myeloid-derived suppressor cells (MDCSs) hold promise in fine-tuning the immune milieu after radiation. Early-phase trials are investigating these strategies, particularly in lung and brain injury, intending to prevent the progression from acute pneumonitis to chronic fibrosis [165–167].

### Regenerative, microenvironment-oriented and mucosal protective therapies

#### Mucosal protection and repair agents

Sucralfate forms a protective coating on ulcerated mucosa, promoting healing. Glutamine supports enterocyte metabolism, while short-chain fatty acids (SCFAs) serve as an energy source for colonic epithelial cells and enhance mucosal immunity. These agents are widely used in the supportive management of RE and RIE, where they reduce pain, diarrhea, and bleeding, and promote epithelial repair [168, 169].

Formalin is a widely known antioxidant and mucosal protectant. It may fix tissues by causing cross-linking of tissue proteins, and it has also been proposed that it may hydrolyze proteins and superficially coagulate tissue to treat intestinal bleeding caused by RE. The use of formalin in the management of RE emerged from its successful use in the treatment of radiation cystitis in 1986 [170]. A meta-analysis of several versions of guidelines for the treatment of RE showed that formalin topical enemas had an 80–100% effective rate for RE. However, there are many adverse effects of this treatment, such as severe local pain, colitis, perforation, stenosis, and ulceration [171].

#### Growth factors

Keratinocyte growth factor (KGF; palifermin) stimulates epithelial proliferation and protects mucosal surfaces. Other agents, such as bFGF and intestinal alkaline phosphatase analogues, enhance epithelial repair and detoxify luminal irritants. They have been trialed mainly for oral mucositis and RE, where they accelerate healing and reduce symptom burden, though concerns about tumor stimulation require ongoing study [172, 173].

#### Cell-based and ecm-targeted therapies

Mesenchymal Stem Cells (MSCs) provide both regenerative and immunomodulatory functions. They are home to injured tissue and secrete trophic factors, including hepatocyte growth factor (HGF), VEGF, and prostaglandin E2 (PGE2). They release antioxidant enzymes such as SOD, suppress Wnt/β-catenin signaling, and upregulate MMPs to remodel the ECM. Importantly, they can induce apoptosis of activated fibroblasts and support epithelial regeneration. Early-phase trials have confirmed the safety and feasibility of MSC infusion in RILI [174]. In RE, preclinical studies demonstrate reduced fibrosis and restored mucosal integrity. In RIBI, MSC transplantation shows neuroprotective effects in animal models [175].

Biomaterials such as hydrogels and oligo-fucoidan act as scaffolds to support tissue regeneration and modulate inflammation. They may restore structural integrity and attenuate fibrosis in irradiated organs [176]. Preclinical and early clinical studies indicate that these drugs demonstrate promising efficacy in RILI and RE [177].

### Physical, technological, and procedural interventions

#### Hyperbaric Oxygen Therapy (HBOT)

HBOT involves breathing 100% oxygen under increased atmospheric pressure, enhancing tissue oxygen delivery. This stimulates angiogenesis, fibroblast activity, and epithelialization, while reducing hypoxia-driven fibrosis and chronic inflammation. Clinically, HBOT is used for refractory radiation proctitis and radiation cystitis, improving bleeding, pain, and healing of necrotic tissue [178].

#### Photobiomodulation (PBM)

Low-level laser or Light-Emitting Diode (LED) therapy activates cytochrome-C oxidase in mitochondria, enhancing Adenosine Triphosphate (ATP) production and triggering anti-inflammatory and pro-regenerative signaling [179]. PBM is increasingly studied for oral mucositis and RD, where it reduces pain and accelerates epithelial healing [180, 181]. Its non-invasive nature and absence of systemic toxicity make it particularly appealing as a supportive therapy.

#### Endoscopic and ablative procedures

Endoscopic argon beam plasma coagulation (APC) is often used in the treatment of gastrointestinal hemorrhage, which uses high-frequency and high-voltage current to ionize argon into an argon plasma beam with strong conductivity, and then the argon plasma beam is directed to the wound tissue (3–5 mm distance) to produce the thermal effect, to realize tissue inactivation and hemostasis. It coagulates the target area without contact with the wound tissues and avoids the adhesion of the catheter head and scab formation after treatment, which can also effectively stop extensive bleeding in a short time [182]. The adverse effects are almost only mild abdominal distension and enterospasm caused by excessive argon blowing. Nowadays, APC therapy has become one of the standard therapies recommended by the clinical treatment guidelines for RE [183].

Furthermore, other treatments, such as Bipolar electrocoagulation, Heater probe, Radiofrequency ablation (RFA), and Cryoablation, have been gradually applied in clinical trials of RE, but have not been included in guidelines due to insufficient samples, uncertified safety, and unclear curative effect [184].

### Emerging therapies and detection methods

#### Microbiota-directed Therapy

IR disrupts the intestinal microbiome, amplifying mucosal injury and systemic inflammation. Probiotics, particularly Lactobacillus and Bifidobacterium strains, reduce diarrhea and inflammation during pelvic radiotherapy by enhancing mucosal barrier integrity and producing anti-inflammatory metabolites [185]. Fecal microbiota transplantation (FMT) provides broader microbial restoration [186].

Early clinical studies confirmed FMT could reduce bleeding and mucosal ulceration in patients with chronic RE. Beyond replacement, engineered bacterial consortia are being developed to produce protective metabolites such as short-chain fatty acids or to deliver therapeutic proteins directly to the irradiated mucosa [187, 188]. These strategies may extend the benefits of microbiome modulation to other organs, such as the lung and brain, through systemic immune signaling [92, 189–191].

#### Senolytics

Radiation generates senescent cells that secrete a pro-inflammatory and profibrotic SASP. Senolytic agents such as navitoclax (ABT-263) or dasatinib plus quercetin selectively eliminate senescent cells, reducing chronic inflammation and fibrosis [192].

Navitoclax, a Bcl-2 family inhibitor, has demonstrated the ability to clear senescent fibroblasts in RILI, resulting in reversal of established fibrosis and improved survival. Similarly, the combination of dasatinib plus quercetin (D + Q) has shown efficacy in preclinical models of RILI and RIHD. Importantly, senolytics differ from classical antifibrotics by directly addressing a fundamental driver of chronic radiation sequelae. Early-phase clinical studies are beginning to evaluate these agents in cancer survivors with prior radiotherapy exposure, marking a major step toward translation [193].

#### Targeted nanomedicine

Nanotechnology introduces unprecedented precision in drug delivery for radiation-induced injury. Nanocarriers, including liposomes, polymeric nanoparticles, and exosomes, can be engineered to concentrate therapeutic agents in irradiated tissues by exploiting enhanced vascular permeability and inflammatory homing signals. This targeted delivery reduces systemic toxicity while increasing local drug concentration.

Inhaled nanoparticles carrying antioxidants, antifibrotics, or anti-inflammatory agents have been tested in animal models, showing reduced radiation-induced alveolar damage and fibrosis. In RE, oral or rectal nanoparticle formulations provide site-specific delivery of cytoprotectants to irradiated mucosa, preserving microbiome balance and mucosal integrity. Exosome-based nanocarriers derived from mesenchymal stromal cells are an especially promising class, as they carry microRNAs and proteins that naturally promote repair. Early translational studies confirm enhanced efficacy and safety compared with free drug formulations. The clinical pipeline now includes nanoparticle-encapsulated Amifostine analogues, liposomal curcumin, and inhaled antioxidant nanomedicines under phase I/II evaluation [194, 195].

#### Exosome therapy (stem-cell-derived extracellular vesicles)

Stem-cell-derived exosomes (EVs) represent a cell-free strategy that delivers microRNAs, proteins, lipids, and antioxidants to irradiated tissues, reducing ROS-driven damage, modulating NF-κB/TGF-β signaling, reprogramming macrophages, and promoting epithelial repair. Adipose-MSC exosomes accelerate skin regeneration via hyaluronan synthase-1, while MSC-exosomes in the lung suppress inflammation and EMT, limiting fibrosis. In the intestine, EVs enhance mucosal healing through Milk Fat Globule-EGF Factor 8 (MFGE8)-phosphatidylserine interactions, and in the brain, neural/MSC-EVs attenuate microglial activation and restore cognition through silent information regulator 1 (SIRT1) and miR-124 pathways [196–198]. Overall, EVs mitigate acute injury and restrain chronic remodeling while avoiding the engraftment risks of live cells.

Preclinical studies support efficacy in RD, RILI, gastrointestinal toxicity, and brain injury [199–201]. Clinically, a phase I trial demonstrated that grape exosomes reduced chemoradiation-related oral mucositis, while additional EV trials remain largely non-radiation but are expanding as manufacturing and quality standards advance [202].

#### Flash radiotherapy (FLASH-RT)

FLASH-RT is an emerging treatment strategy that delivers ultra-high-dose radiation at exceptionally fast delivery rates, typically exceeding 10 Gy per fraction. Studies indicate that the rapid delivery of radiation induces a distinct biological response, including reduced oxidative stress and inflammation, compared to conventional radiotherapy. Preclinical studies have shown that FLASH-RT reduces radiation-induced damage to normal tissues, such as the lungs, gastrointestinal tract, and skin, by protecting these tissues from inflammation and fibrosis. In contrast, tumor cells remain highly susceptible to high-dose radiation, and evidence suggests that the efficacy of FLASH-RT in treating tumors may be comparable to or even surpass conventional radiotherapy [203–205].

Early-phase clinical trials are underway to assess the safety and efficacy of FLASH-RT in humans. The first clinical trial of FLASH-RT in cancer patients was conducted in 2019, and since then, several trials have been launched to evaluate its application in various cancers, including non-small cell lung cancer and soft tissue sarcomas [206, 207]. Preliminary results from these trials indicate that FLASH-RT is well-tolerated, with promising reductions in normal tissue toxicity without compromising tumor control. However, the clinical application of FLASH-RT remains in the early stages, and further extensive trials are necessary to establish optimal dosing, treatment protocols, and long-term outcomes [208].

Additionally, complementary treatments, including those based on traditional Chinese medicine, topical synthetic agents, and protein/polysaccharide bioengineered scaffolds, are showing increasing efficacy in mitigating radiation-induced damage, particularly in the gastrointestinal tract and skin [209–214]. These therapies not only aim to alleviate symptoms but also target the underlying biological mechanisms of radiation-induced injury at the molecular and cellular levels (Table 1).

**Table 1:** Ongoing or completed drugs in clinical trials

Interventional Type	Drugs	Mechanism Of Action	ID	Status	Phase
**Radiation-induced Lung Injury**					
Glucocorticoids (GCs)	Methylprednisolone	Reduce inflammation by suppressing immune responses and cytokine production	NCT03661567	Completed	II
	Prophylactic Inhaled Steroids		NCT03803787	Recruiting	II
Antioxidants	Amifostine	Scavenge free radicals, reducing DNA damage	NCT00130143	Completed	Not Applicable
			NCT00004176	Completed	II
			NCT00003583	Unknown	II
			NCT00003089	Completed	II
			NCT00158041	Completed	IV
			NCT00003313	Completed	III
			DOI:https://doi.org/10.1016/S0360-3016(01)01713-8	Completed	III
	Topical Superoxide Dismutase (SOD)	Neutralize ROS, reducing oxidative stress and preventing cellular damage	NCT01771991	Completed	Not Applicable
	Glutathione (GSH)	Protect cells from oxidative stress by neutralizing free radicals	DOI: https://doi.org/10.3969/j.issn.1672-9455.2018.2018.01.027	Completed	II
	Metformin	Activate AMPK, reduce oxidative stress, and enhance repair	ChiCTR1900026208	Pending	Not Applicable
RAAS System Inhibitor	Losartan	Reduce inflammation, fibrosis, and oxidative stress. Reduce the stimulation of TGF-β signaling	NCT05637216	Active, not recruiting	II
	Losartan		NCT00880386	Withdrawn	Not Applicable
	Valsartan		ChiCTR2200062762	Completed	IV
	Angiotensin-converting enzyme inhibitors (ACEI)		DOI:https://doi.org/10.1097/COC.0000000000000324	Completed	III
TGF-β Receptor Inhibitor	Pirfenidone	Reduce fibrosis and inflammation, promote tissue regeneration, and improve repair processes	NCT03902509	Completed	II
			NCT02296281	Unknown	II
			NCT05801133	Recruiting	II
			NCT05704166	Recruiting	II
			ChiCTR2200064594	Recruiting	II
			CTRI/2021/07/035223	Active, not recruiting	II
			ChiCTR2100043032	Recruiting	IV
			NCT00020631	Completed	Not Applicable
VEGF Receptor Inhibitor	Bevacizumab (Avastin)	Block angiogenesis, reduce vascular permeability, and limit radiation-induced tissue damage and fibrosis	NCT01917877	Recruiting	II
	Endostatin		NCT03796364	Completed	II
HMG-CoA reductase inhibitor	Ulinastatin	Reduce inflammation and oxidative stress, promote tissue repair	DOI:https://doi.org/10.1007/s12032-014-0405-x	Completed	III
Tyrosine Kinase Inhibitor	Nintedanib	Inhibit RhoA-ROCK signaling, reducing inflammation and fibrosis, and promoting tissue repair	NCT02452463	Completed	II
Mesenchymal stem cells (MSCs)	Umbilical Cord MSCs	Promote tissue repair, reduce inflammation, and enhance regeneration	NCT02277145	Completed	I
	Umbilical Cord MSC		ChiCTR1800019309	Pending	I + II
Others	Pentoxifylline (PTX) + Vitamin E	Reduce oxidative stress, improve tissue oxygenation, and mitigate radiation-induced fibrosis	NCT01871454	Recruiting	II
	PTX-tocopherol and Hyperbaric Oxygen		NCT01822405	Recruiting	II
	Oligo-Fucoidan	ECM analog. Promote tissue regeneration, enhance wound healing, and reduce fibrosis	NCT05616507	Recruiting	Not Applicable
	Pemetrexed	Inhibit folate metabolism, reducing inflammation and promoting tissue repair	ChiCTR-TRC-13004184	Completed	Not Applicable
	Traditional Chinese medicine	Modulate immune responses, reduce inflammation, and promote tissue repair	ChiCTR2200063252	Pending	II
			ChiCTR2300073364	Recruiting	IV
			ChiCTR2300072148	Recruiting	II
			ChiCTR2000033285	Completed	II
			ChiCTR2300068524	Recruiting	IV
**Radiation Enteritis**					
GCs	Glucocorticoid	Reduce inflammation by suppressing immune responses and cytokine production	NCT06410443	Recruiting	Not Applicable
	Beclomethasone		NCT01073384	Completed	I + II
Antioxidants	N-acetyl Cysteine (NAC) and Zinc	Reduce oxidative stress, scavenge free radicals	NCT06482034	Recruiting	II
	NAC		NCT06354712	Recruiting	II
	α-Lipoic Acid	Reduce oxidative stress, enhance antioxidant defense	NCT05023863	Unknown	II + III
	Amifostine	Scavenge free radicals, reducing DNA damage	NCT01586117	Unknown	II
			NCT00003580	Completed	II
			NCT00025298	Terminated	II
	GC4419	A selective superoxide dismutase mimetic that reduces oxidative stress	NCT02508389	Completed	II
			NCT04819685	Recruiting	IV
	Kang Fu Pen		NCT03689712	Completed	III
	Vitamin E and Hydrogen-Rich Water	Reduce oxidative stress, improve tissue oxygenation, and mitigate radiation-induced fibrosis	NCT04713332	Unknown	III
	PTX + Vitamin E		NCT02397486	Completed	II
	Vitamin D		NCT04308161	Unknown	II
	Melatonin	Activate AMPK, reduce oxidative stress, and enhance repair	NCT03833570	Completed	II
	Ulinastatin	Inhibit proteases, reduce inflammation	NCT03387774	Active, not recruiting	III
	Tempol	Reduce oxidative stress, scavenge free radicals	NCT03480971	Unknown	II
	Tetrahydrobiopterin (BH4)	Reduce oxidative stress, enhance nitric oxide production, and support tissue repair	NCT05138887	Unknown	II
Anti-inflammatory Drugs	Benzydamine	Inhibit COXs, reducing inflammation, and alleviating pain	NCT05055726	Completed	IV
			NCT00051441	Completed	III
	Celecoxib		NCT00698204	Completed	II
	Celecoxib and Erlotinib		NCT00970502	Completed	I + II
	Thalidomide combined with Glutamine	Target TNF-α, reducing inflammation, and promoting tissue repair	NCT06031012	Not yet recruiting	III
	Edible Plant Exosome	Deliver bioactive molecules, reduce inflammation, and promote tissue repair	NCT01668849	Completed	I
	Hyperimmune Colostrum	Boost immune response, reduce inflammation, and enhance tissue repair	NCT00699569	Unknown	Not Applicable
	TRAUMEEL S	Reduce inflammation, promote tissue repair, and accelerate healing	NCT00584597	Completed	I
	Brilacidin	A small-molecule mimic of antimicrobial peptides, modulating immune responses and reducing inflammation	NCT02324335	Completed	II
MSCs	Systemic MSC	Promote tissue repair, reduce inflammation, and enhance regeneration	NCT02814864	Unknown	II
	MSC		NCT06599346	Recruiting	Not Applicable
	Umbilical Cord MSC(TH-SC01)		NCT05939778	Recruiting	I
	Autologous regenerative cells of adipose tissue		NCT03643614	Completed	I
Microbiota-related therapy	Fecal microbiota transplantation (FMT)	Restore gut microbiota balance, reduce inflammation, and enhance intestinal barrier function	NCT03516461	Unknown	Not Applicable
	Lactobacillus Plantarum 299v		NCT06019312	Recruiting	Not Applicable
	Prebiotic Enhanced Diet		NCT01414517	Unknown	III
	Probiotics		NCT05032027	Recruiting	Not Applicable
			NCT06122636	Enrolling by invitation	Not Applicable
			NCT01473290	Withdrawn	III
			NCT01839721	Completed	III
			NCT03978949	Unknown	III
			NCT05032027	Recruiting	Not Applicable
			NCT01839721	Completed	III
Hyperbaric Oxygen Therapy (HBOT)	HBOT	Increase oxygen supply, enhance tissue repair, reduce inflammation, and promote healing	NCT00134628	Terminated	III
HMG-CoA reductase inhibitor	Lovastatin	Reduce inflammation and oxidative stress, promote tissue repair	NCT00580970	Completed	II
Mucosal Protectant	Magic Mouthwash Plus Sucralfate	Form a protective barrier, reducing inflammation and promoting healing	NCT00814359	Completed	III
	Pentosan Polysulfate		NCT00003825	Completed	III
	Omega-3 Hydrogel		NCT05214495	Completed	II + III
	Aloe Vera Gel and Manuka Honey		NCT06381635		
	Manuka Honey		NCT00615420		
	Manuka Honey	Reduce oxidative stress, enhance anti-inflammatory responses, and promote healing	NCT01262560		
	Honey		NCT01465308		
	Coconut Oil		NCT03176368	Unknown	Not Applicable
	Olive Oil		NCT05322421	Completed	Not Applicable
	East Indian Sandalwood Oil (EISO)		NCT02399228	Completed	II
	Deep-sea Fish Oil		NCT06392971	Recruiting	II
	Solcoseryl and pumpkin seed oil		NCT04303312	Unknown	III
	Propolis	Form a protective barrier to reduce inflammation. Enhance antioxidant activity and promote tissue regeneration	NCT01375088	Completed	II
	Episil®		NCT03546985	Completed	Not Applicable
	Tepilta®		NCT01336530	Terminated	III
	Orosol®		NCT05161091	Recruiting	Not Applicable
	Homeodent®		NCT01066741	Terminated	III
	FORRAD®		NCT02735317	Unknown	II
	SAMITAL®		NCT01941992	Completed	II
Nutritional Management	Nutritional Management	Enhance gut barrier function, support intestinal epithelial cell regeneration, and reduce inflammation	NCT05721885	Not yet recruiting	Not Applicable
	Capsules containing dietary fiber		NCT04534075	Recruiting	III
	Aloe Vera Juice		NCT05369234	Completed	III
	Hyaluronic Acid		NCT06469216	Recruiting	I + II
	Thalidomide combined with Glutamine		NCT06617182	Active, not recruiting	II
	Glutamine		NCT05856188	Recruiting	Not Applicable
			NCT00828399	Completed	IV
			NCT01758783	Unknown	Not Applicable
			NCT00006994	Terminated	III
			NCT03015077	Completed	Not Applicable
Growth factors	SYN-020 (recombinant bovine Intestinal Alkaline Phosphatase)	Stimulate cell proliferation, tissue regeneration, and enhance recovery	NCT05045833	Completed	I
			NCT04815993	Completed	I
	Recombinant Bovine Basic Fibroblast Growth Factor		NCT03778008	Unknown	II
	Keratinocyte growth factor		NCT00004132	Completed	II
	Palifermin		NCT00728585	Withdrawn	II
Small Molecule Inhibitors	TK-112690 (Uridine phosphorylase inhibitor)	Reduce DNA damage, improve cellular repair processes, and limit radiation-induced fibrosis and inflammation	NCT05658016	Active, not recruiting	II
	Octreotide	Inhibit growth hormone release, reduce inflammation	NCT00075868	Completed	III
	Esomeprazole	Inhibit proton pumps, reducing gastric acid secretion	NCT06120803	Recruiting	II
	Gabapentin	Modulate calcium channels, reducing nerve pain and inflammation	NCT02480114	Completed	III
			NCT03269344	Completed	III
Physical therapy	Photobiomodulation (PBM)	Promote tissue repair, reduce inflammation, and enhance cellular regeneration	NCT04671862	Recruiting	Not Applicable
			NCT06458517	Not yet recruiting	Not Applicable
	LED PBM		NCT04251949	Completed	II
	Low-Level Laser Therapy	Stimulate cellular repair, reduce inflammation, and promote tissue regeneration	NCT02604329	Completed	Not Applicable
			NCT03955224	Withdrawn	II
			NCT01876407	Unknown	IV
Surgery	CryoSpray Ablation (TM)	Induce tissue cooling, reducing inflammation and promoting healing	NCT00756197	Withdrawn	IV
	Surgery	Remove damaged tissue, alleviate complications, and promote healing	NCT05607927	Not yet recruiting	III
	Drug Retention Enema	Deliver therapeutic agents directly to the intestine, reducing inflammation and promoting healing	NCT06325982	Recruiting	Not Applicable
Others	Capsaicin Lozenges	Reduce pain and inflammation by desensitizing sensory neurons	NCT00003610	Completed	III
	Ectoin®	Stabilize cell membranes, protect tissues from radiation-induced oxidative damage	NCT03932292	Completed	Not Applicable
	Traditional Chinese medicine	Modulate immune responses, reduce inflammation, and promote tissue repair	NCT04204382	Unknown	IV
			NCT04888234	Unknown	II
			NCT05040425	Unknown	II
			NCT01898091	Completed	II
	Dexpanthenol	A derivative of the vitamin B group, promoting epithelial cell regeneration, reducing inflammation, and enhancing healing	NCT01318889	Unknown	II
	IZN-6N4	A small molecule drug, reducing oxidative stress, modulating inflammation, and promoting tissue repair	NCT01400620	Completed	II
	Loperamide and Tincture of Opium—(Loop)	An opioid receptor, targeting the μ-opioid receptors in the gastrointestinal tract, regulates bowel movements and alleviates damage	NCT00444093	Terminated	III
**Radiation-induced Brain Injury**					
Neuroprotective drugs	Memantine (NMDAR antagonist) and Pioglitazone (PPAR-γ agonist)	Reduce excitotoxicity and neuronal damage caused by excessive glutamate release, which is often triggered by radiation. Reduce inflammation, oxidative stress, and fibrosis	NCT06594172	Not yet recruiting	II
	Memantine		NCT00566852	Completed	III
	Armodafinil	Enhance alertness and reduce fatigue by modulating neurotransmitters, aiding recovery	NCT01032200	Completed	II
	Donepezil	Inhibit acetylcholinesterase, enhancing neurotransmission and potentially improving cognitive function	NCT00452868	Completed	I
	lithium carbonate	Modulate neuroinflammation, enhance cellular repair, and protect against radiation-induced cognitive decline	NCT00469937	Terminated	I
Antioxidants	Edaravone	Scavenge free radicals, reduce oxidative stress	NCT01865201	Completed	II
	Pioglitazone Hydrochloride	Activate PPAR-γ, reducing inflammation, oxidative stress, and promoting tissue repair	NCT01151670	Completed	I
RAAS System Inhibitor	Ramipril	Reduce inflammation, fibrosis, and oxidative stress. Reduce the stimulation of TGF-β signaling	NCT03475186	Active, not recruiting	II
VEGF Receptor Inhibitor	Apatinib	Block angiogenesis, reduce vascular permeability, and minimize radiation-induced tissue damage and fibrosis	NCT04152681	Unknown	II
	Bevacizumab		NCT01621880	Completed	II
			NCT05303259	Recruiting	II
	Endostar combined with Corticosteroid		NCT05177237	Recruiting	II
Immunomodulator	Thalidomide	Inhibit TNF-α, reducing inflammation and promoting tissue repair	NCT03208413	Unknown	II
**Radiation Dermatitis**					
Antioxidants	Trental & Vitamin E	Reduce oxidative stress, improve tissue oxygenation, and mitigate radiation-induced fibrosis	NCT00583700	Completed	II
	PTX + Vitamin E		NCT02898376	Unknown	III
	BH4	Reduce oxidative stress, enhance nitric oxide production, and support tissue repair	NCT05114226	Unknown	I
			NCT05299203	Unknown	I
Natural agents	Curcumin	Reduce oxidative stress, modulate inflammation, and promote tissue healing	NCT01246973	Completed	II + III
			NCT01042938	Completed	II
	Curcumin Gel		NCT05982197	Completed	Not Applicable
			NCT02556632	Completed	II
	Nigella sativa	Reduce inflammation, scavenge free radicals, and enhance tissue repair	NCT05693597	Completed	II
	Colchicine	Inhibit microtubule polymerization, reducing inflammation and fibrosis	NCT05335148	Recruiting	I
	Tahini	Reduce inflammation, support antioxidant defenses, and promote tissue repair	NCT04890197	Unknown	Not Applicable
	Grape juice	Reduce oxidative stress, scavenge free radicals, and promote tissue healing	NCT04890184	Unknown	Not Applicable
	Aloe Vera Gel	Reduce inflammation, promote tissue healing, and accelerate recovery	NCT00876642	Completed	III
	Bacterial Cellulose-monolaurin Hydrogel	reduces inflammation, Support tissue regeneration	NCT05079763	Unknown	II
	Herb extracts	Reduce inflammation, enhance antioxidant activity, and promote tissue repair	NCT03359187	Completed	Not Applicable
			NCT02922244	Completed	Not Applicable
Topical Synthetic Agents	Norepinephrine (NE)	Target adrenergic receptors, modulating vascular tone, reducing inflammation, and promoting tissue repair	NCT01367990	Terminated	I
	Jalosome® Soothing Gel. (JALOSOME-01)	Enhance skin hydration, reduce inflammation, and promote healing	NCT05284487	Recruiting	Not Applicable
	APN201 (Liposomal Recombinant Human Cu/Zn-Superoxide Dismutase)	Scavenge superoxide radicals, reduce oxidative stress	NCT01513278	Completed	I + II
	ST266	Modulate immune responses, reduce inflammation, and promote tissue repair	NCT01714973	Completed	I
	DermoRelizemaTM cream	Reduce inflammation, soothe irritation, and promote healing	NCT04483856	Completed	Not Applicable
	LUT014	Reduce oxidative stress, modulate inflammation, and promote tissue repair	NCT04261387	Completed	II
	Radiation Care® Gel	Soothe skin, reduce inflammation, and promote healing	NCT04995328	Completed	Not Applicable
	KeraStat Cream	Enhance skin barrier function, reduce inflammation, and accelerate healing	NCT03374995	Completed	Not Applicable
	Gelronate Gel vs. Aloevera	Target tissue regeneration by promoting collagen synthesis, reducing inflammation, or providing antioxidant protection	NCT03941665	Completed	Not Applicable
	Modified Dakin's Solution	Act as a disinfectant, reducing infection risk, promoting wound healing, and preventing inflammation	NCT02369835	Completed	III
	Mebo ointment	Promote tissue regeneration, reduce inflammation, and enhance healing	NCT06117904	Completed	Not Applicable
	Mepitel Film	Act as a soft silicone dressing that protects irradiated skin, reduce pain, prevent infection, and support tissue healing	NCT03910595	Completed	Not Applicable
	Hydrosorb®	A hydrocolloid dressing that absorbs exudates, maintains a moist wound environment, reduces pain, and accelerates healing	NCT02839473	Completed	III
	Alantel®	Reduce inflammation, soothe skin irritation, and promote tissue regeneration	NCT04116151	Completed	Not Applicable
	StrataXRT	A silicone-based gel that forms a protective barrier over irradiated skin, reducing inflammation, promoting tissue healing	NCT05073172	Withdrawn	Not Applicable
	Biafine Cream	Enhance skin hydration, reduce inflammation, and promote tissue healing	NCT00006481	Completed	III
	Doxepin	A tricyclic antidepressant that inhibits histamine receptors, reducing itching and inflammation	NCT02447211	Unknown	II
	Fenofibrate	Activate PPAR-α, improve lipid metabolism, and reduce inflammatory responses	NCT03557983	Unknown	Not Applicable
	OTD70DERM	Reduce inflammation and oxidative stress. Promote wound healing and enhance tissue regeneration	NCT01228565	Completed	III
	Xonrid®		NCT03255980	Completed	Not Applicable
			NCT02261181	Completed	Not Applicable
	MTS-01		NCT00713154	Completed	II
	Tempol	Reduce oxidative stress, scavenge free radicals	NCT00801086	Unknown	II
Small Molecule Inhibitors	JBM-TC4 (Bruton`s tyrosine kinase (BTK) inhibitor)	Inhibit BTK, reducing inflammatory responses and immune cell activation	NCT02289365	Unknown	II
Physical therapy	Silver Leaf Dressing	Create a protective barrier, enhancing tissue regeneration, and reducing oxidative stress	NCT00207324	Completed	III
	FR-101 chest dressing		NCT06040983	Not yet recruiting	Not Applicable
	Pulsed Electromagnetic Field Therapy	Stimulate cellular repair, enhance circulation, reduce inflammation, and accelerate tissue regeneration	NCT06003764	Completed	Not Applicable
	Laser Therapy	Stimulate cellular metabolism, reduce inflammation, enhance collagen production, and improve blood flow	NCT02384434	Completed	Not Applicable
			NCT01932073	Completed	Not Applicable
	Manual Therapy	Improve circulation, reduce inflammation, alleviate pain, and enhance tissue healing	NCT04850170	Recruiting	Not Applicable
Surgery	Adipose-Induced Regeneration of Breast Skin (AIR Breast)	Utilize adipose-derived stem cells to promote tissue regeneration, enhance collagen production, reduce inflammation, and stimulate healing	NCT03981718	Withdrawn	Not Applicable
**Radiation-induced bone injury**					
GCs	Dexamethasone	Reduce inflammation by suppressing immune responses and cytokine production	NCT01248585	Completed	III

Data sources: Clinical trial registration website, Chinese clinical trial registry website

The integration of various therapeutic strategies offers promising potential for developing more effective countermeasures against radiation-induced injury [215, 216]. The future of radiation-induced injury treatment lies in the ability to tailor therapies to individual patient profiles and the specific mechanisms of injury, ensuring both optimal efficacy and minimal side effects.

## Discussion and future perspectives

Radiation-induced injury is a dynamic, multifaceted pathology, although over recent decades, substantial progress has clarified the cellular and molecular underpinnings of radiation-induced injury, important gaps persist. Much of the existing research is fragmented, with studies often focused on individual organs or pathways rather than considering radiation-induced injury as a systemic and dynamic process. While the roles of DNA DSBs, oxidative stress, and chronic inflammation are well understood, a unified framework that incorporates epigenetic regulation, mitochondrial dysfunction, and stromal-immune interactions is still lacking. Clinical translation is also hindered by studies with short follow-up periods and inconsistent exposure protocols. Finally, inter-individual variability, which is shaped by genetics, age, comorbidities, and the microbiome, remains insufficiently defined.

Future progress will require a shift toward proactive, mechanism-based, and patient-tailored strategies. Precision-medicine frameworks should harness multi-omic profilings, single-cell transcriptomics, spatial proteomics, epigenomics, along with advanced imaging and computational modeling, to define biomarkers that predict organ sensitivity, disease trajectory, and treatment response. Emphasis should be placed on inter-organ signaling networks, including circulating cytokines, extracellular vesicles, and immune cell trafficking, which influence systemic effects. Emerging platforms, such as CRISPR-based screens, organoids, and humanized animal models, offer powerful means to probe causality and evaluate interventions with greater translational fidelity. Integrating systems biology with clinical radiotherapy data can bridge preclinical discoveries to patient outcomes. Addressing these gaps will move the field from description to actionable mechanisms, enabling durable countermeasures that mitigate acute injury and restore long-term organ function. International, multicenter studies and standardized databases are also necessary to capture epidemiological data and patient variability, paving the way for personalized treatments. The next era of radiation biology should thus prioritize holistic, predictive, and regenerative solutions with direct clinical impact.

## Data Availability

The data that support the findings of this study are available from the corresponding author upon reasonable request.
